# Superoxide-mediated phosphorylation and stabilization of Mcl-1 by AKT underlie venetoclax resistance in hematologic malignancies

**DOI:** 10.1038/s41375-025-02694-4

**Published:** 2025-07-24

**Authors:** Stephen J. F. Chong, Jolin X. H. Lai, Kartini Iskandar, Benedict J. Leong, Chuqi Wang, Yuhan Wang, Romain Guièze, Deepika Raman, Rachel H. F. Lim, Catherine J. Wu, Wee Joo Chng, Alice M. S. Cheung, Charles Chuah, Matthew S. Davids, Shazib Pervaiz

**Affiliations:** 1 Department of Physiology, Yong Loo Lin School of Medicine, National University of Singapore (NUS), Singapore, Singapore; 2 Cancer Science Institute of Singapore, NUS, Singapore, Singapore; 3 NUS Centre for Cancer Research (N2CR), Yong Loo Lin School of Medicine, NUS, Singapore, Singapore; 4 Department of Medical Oncology, Dana-Farber Cancer Institute, Boston, MA USA; 5 Department of Pharmacy and Pharmaceutical Sciences, NUS, Singapore, Singapore; 6 Department of Cell Therapy and Clinical Hematology, University Hospital of Clermont-Ferrand, University Clermont Auvergne, Clermont-Ferrand, France; 7 Department of Haematology, Singapore General Hospital and National Cancer Centre Singapore, Singapore, Singapore; 8 National University Cancer Institute, National University Health System, Singapore, Singapore; 9 SingHealth Duke-NUS Medicine Academic Clinical Programme, Duke-NUS Medical School, Singapore, Singapore; 10 Cancer and Stem Cell Biology Program, Duke-NUS Medical School, Singapore, Singapore; 11 Integrative Science and Engineering Programme (ISEP), NUS Graduate School, NUS, Singapore, Singapore; 12 NUS Medicine Healthy Longevity Program, NUS, Singapore, Singapore

**Keywords:** Acute myeloid leukaemia, Cancer therapeutic resistance

## Abstract

Resistance to the Bcl-2-specific inhibitor, Venetoclax (VEN), poses a therapeutic challenge in the management of chronic lymphocytic leukemia and acute myeloid leukemia. Although VEN resistance has been linked to Mcl-1 upregulation, thereby switching survival dependence from Bcl-2 to Mcl-1, the mechanism underlying increased Mcl-1 expression remains elusive. Given that changes in cellular redox state affect cancer cell fate, we investigated the crosstalk between intracellular redox milieu and Mcl-1 upregulation in VEN-resistant cells. Results show that increased Mcl-1 protein levels in VEN-resistant hematologic malignant cells are associated with elevated intracellular superoxide (O_2_^.−^) levels. Validating that, augmenting intracellular O_2_^.−^ in VEN-sensitive cells increases Mcl-1 phosphorylation at threonine-163 (T163pMcl-1) and protein stability via reduced Mcl-1 ubiquitination and degradation. Furthermore, redox-activated AKT/PKB is implicated in O_2_^.−^-induced T163pMcl-1, as reducing intracellular O_2_^.−^ or inhibiting AKT significantly decreases T163pMcl-1 and Mcl-1 accumulation, which amplifies mitochondrial apoptotic priming and restores VEN sensitivity. Importantly, combination therapy with AKT inhibitor, capivasertib, and VEN reduced VEN-resistant cells systemically and prolonged survival in a murine model. Collectively, a novel redox-dependent mechanism of Mcl-1 stability is demonstrated for the acquisition of VEN resistance, which has therapeutic implications for employing redox modulating strategies and AKT inhibitors against VEN-resistant hematologic malignancies.

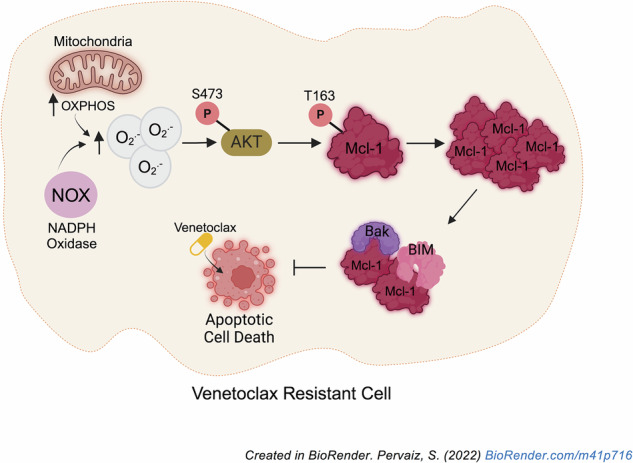

## Introduction

Apoptosis evasion is a cancer hallmark, mediated by aberrant expression and survival dependence on anti-apoptotic members of the Bcl-2 family (Bcl-2, Bcl-xL, and Mcl-1), which function to sequester pro-apoptotic proteins (Bak and Bax). As such, the development of selective anti-apoptotic Bcl-2 inhibitors was explored to reinstate apoptotic functions [1, 2].

Venetoclax/ABT199 (VEN) is a Bcl-2-specific inhibitor, clinically approved for the treatment of chronic lymphocytic leukemia (CLL) and acute myeloid leukemia (AML) [1, 3, 4]. While VEN triggers apoptosis in Bcl-2-dependent hematological cancers, other anti-apoptotic proteins such as Mcl-1 or Bcl-xL appear to play an important role(s) in VEN responsiveness [5, 6]. Prolonged VEN therapy has been shown to reduce survival dependence of hematological cancers on Bcl-2, thereby promoting the selective survival of VEN-refractory cells [7, 8]. While reports have linked *BCL2* mutation(s), such as the *G101V* to inefficient VEN binding to the BH3 domain of Bcl-2 [9–11], this is believed to be a rare occurrence with low allelic fraction [9, 10]. A probable mechanism underlying VEN resistance is the upregulation of the anti-apoptotic protein Mcl-1 [7, 12–15]. Cells with higher Mcl-1 levels are resistant to VEN treatment [7, 16], while inhibiting Mcl-1 restores sensitivity of VEN-R cells to VEN, thus indicating a switch of dependence from Bcl-2 to Mcl-1 [13, 17, 18]. Nonetheless, the underlying mechanism of Mcl-1 upregulation in VEN-R cells has not been well-established to date.

Mcl-1 was first identified in human myeloblastic leukemia and shown to have anti-apoptotic and other physiological functions. Mcl-1 is a short-lived protein, with its expression regulated at multiple cellular levels. Structurally, besides the Bcl-2 homology (BH)1-BH4 domains, Mcl-1 also contains a PEST domain in the N-terminus that functions as signal for protein turnover [19]. Mcl-1 stability is regulated by several ubiquitination and phosphorylation sites [20]. Threonine-163 (T163) phosphorylation stabilizes Mcl-1, while multisite phosphorylation (T92, S121, S159) promotes Mcl-1 degradation [21–26]. Notably, the complex nature of its regulation, coupled with non-apoptotic functions, such as in cardiomyocyte physiology [27], impact the systemic use of Mcl-1 inhibitors. Therefore, a better understanding of the mechanism(s) and/or signaling networks involved in regulating Mcl-1 stability could have potential therapeutic implications, particularly in the setting of VEN resistance with a switch to Mcl-1 dependence.

While the canonical anti-apoptotic activity of the Bcl-2 proteins involves mediating mitochondria outer membrane permeabilization (MOMP), our work has linked altered cellular redox status as the non-canonical pro-survival mechanism [28], such as increased mitochondrial oxygen consumption and intracellular superoxide (O_2_^.−^) levels upon overexpression of Bcl-2 [29–31]. Reduction of intracellular O_2_^.−^ restored sensitivity of Bcl-2-expressing cells to drug-induced apoptosis. Mechanistically, elevated intracellular O_2_^.−^ resulted in sustained phosphorylation of Bcl-2 at serine-70 (S70pBcl-2) via redox-dependent inactivation of the protein phosphatase-2A (PP2A) [32, 33].

Against the backdrop of the crosstalk between cellular redox state and the anti-apoptotic activity of Bcl-2, we questioned the role of cellular redox milieu in the acquisition of VEN-R and the associated increase in Mcl-1 levels. Herein, we provide evidence linking an increase in intracellular O_2_^.−^ to Mcl-1 abundance in VEN-resistant (VEN-R) hematologic malignant cells. Furthermore, O_2_^.−^-induced Mcl-1 stability is a function of AKT-mediated T163pMcl-1, decreased overall mitochondrial priming and increased cell survival.

## Results

### Venetoclax resistance is associated with increased intracellular O_2_^.−^

**Fig. 1:**
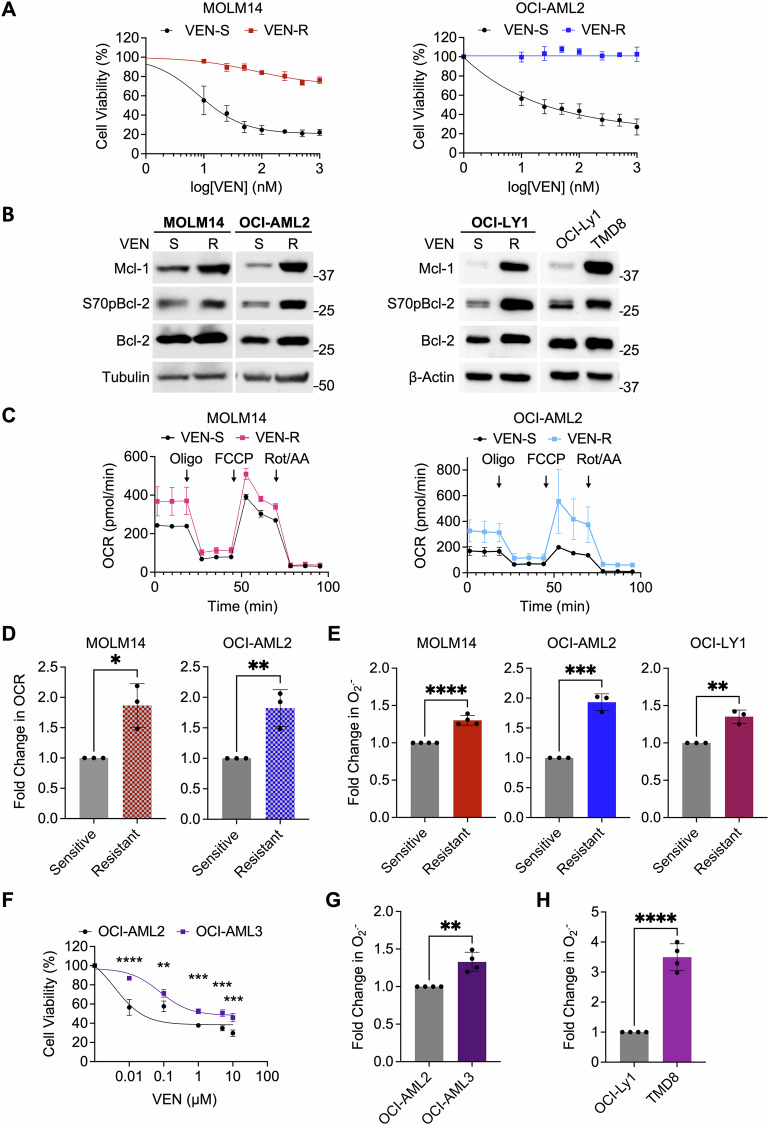
Acquired venetoclax-resistant hematologic cell lines possess higher intracellular O_2_^.−^ and Mcl-1 levels. **A** Cell viability of venetoclax-sensitive (VEN-S) MOLM14, OCI-AML2, and acquired venetoclax-resistant (VEN-R) MOLM14 and OCI-AML2 cells following treatment with increasing concentration of venetoclax (VEN) for 48 h. Cell viability was measured using MTT assay. *N* = 3. **B** Western blot analyses showing Mcl-1, S70pBcl-2, Bcl-2, and Tubulin/β-Actin levels in MOLM14, OCI-AML2, OCI-Ly1 of VEN-S/parental or VEN-R cells, and TMD8 (inherently resistant) cells. *N* = 3. **C** Representative graph showing the kinetics of oxygen consumption rate (OCR) when MOLM14 and OCI-AML2 of VEN-S and VEN-R cells were subjected to oligomycin (1 µM), FCCP (1 µM), and rotenone/antimycin A (Rot/AA) (0.5 µM/0.5 µM) injection at the indicated timepoints of the assay. **D** Graph showing fold change in oxygen consumption rate (OCR) of MOLM14 and OCI-AML2 of VEN-S and VEN-R cells. OCR was measured via Seahorse assay (Agilent, USA). *N* = 3. Unpaired *t*-test was used. **E** Graph showing fold change in intracellular O_2_^.−^ levels of MOLM14 (*N* = 4), OCI-AML2 (*N* = 3), OCI-Ly1 (*N* = 3) of VEN-S and VEN-R cells. Unpaired *t*-test was used. **F** Cell viability of VEN-S OCI-AML2 and inherently VEN-R OCI-AML3 cells following treatment with increasing concentrations of VEN (µM) for 48 h. Cell viability was measured via MTT assay. *N* = 3. Sidak’s multiple comparisons test was used. **G**-**H** Graph showing fold change in intracellular O_2_^.−^ levels of inherently VEN-R OCI-AML3 or TMD8 cells relative to VEN-S OCI-AML2 or OCI-Ly1 cells, respectively. *N* = 4. Unpaired *t*-test was used. *MOLM14 and VEN-S MOLM14 or OCI-Ly1 and VEN-S OCI-Ly1 are used interchangeably.

To study the interplay between intracellular redox state and Mcl-1 upregulation upon the acquisition of VEN resistance, we generated the VEN-R AML cells (VEN-R MOLM14 and VEN-R OCI-AML2) from VEN-sensitive or parental (VEN-S) MOLM14 and OCI-AML2 cells (Figs. 1A, S1A, Supplementary Method). Consistent with published reports [5, 15], we verified Mcl-1 abundance in the acquired VEN-R cells (R) compared to the VEN-S (S) cells of AML as well as in our previously established lymphoid VEN-R diffused large B-Cell lymphoma (DLBCL) cells (Fig. 1B) [7, 13]. Similarly, our previously characterized inherent VEN-R DLBCL cells, TMD8, in comparison to the VEN-S OCI-Ly1 cells [7], showed increased Mcl-1 levels (Fig. 1B). As previously described in VEN-R lymphoid malignancies [7], we also observed an increase in S70pBcl-2 in these VEN-R AML cells (Fig. 1B), indicative of an altered cellular redox milieu [32]. As Mcl-1 regulates apoptosis and mitochondrial bioenergetics [34], we further examined the oxidative phosphorylation (OXPHOS) status of VEN-R cells. Both VEN-R cell lines exhibited higher basal oxygen consumption rate (OCR) and maximal respiration rate compared to VEN-S cells (Fig. 1C, D). Given that enhanced OXPHOS has been linked to the generation of a more pro-oxidant and drug-resistant milieu [13, 35], we observed a significant increase in intracellular O_2_^.−^ in acquired VEN-R MOLM14, OCI-AML2, and OCI-Ly1 cells (Fig. 1E). Corroborating the latter, inherently VEN-R OCI-AML3 (relative to VEN-S OCI-AML2, Fig. 1F) and TMD8 cells also exhibited higher intracellular O_2_^.−^ compared to VEN-S OCI-AML2 and OCI-Ly1 cells, respectively (Fig. 1G, H). Importantly, treatment with VEN for 48 h could also increase reactive oxygen species (ROS) in VEN-S MOLM14 cells (Fig. S2A). Collectively, these data suggest an interplay between intracellular redox state and Mcl-1 accumulation upon VEN resistance acquisition.

### Intracellular O_2_^.−^ increases Mcl-1 and reduces mitochondrial apoptotic priming

**Fig. 2:**
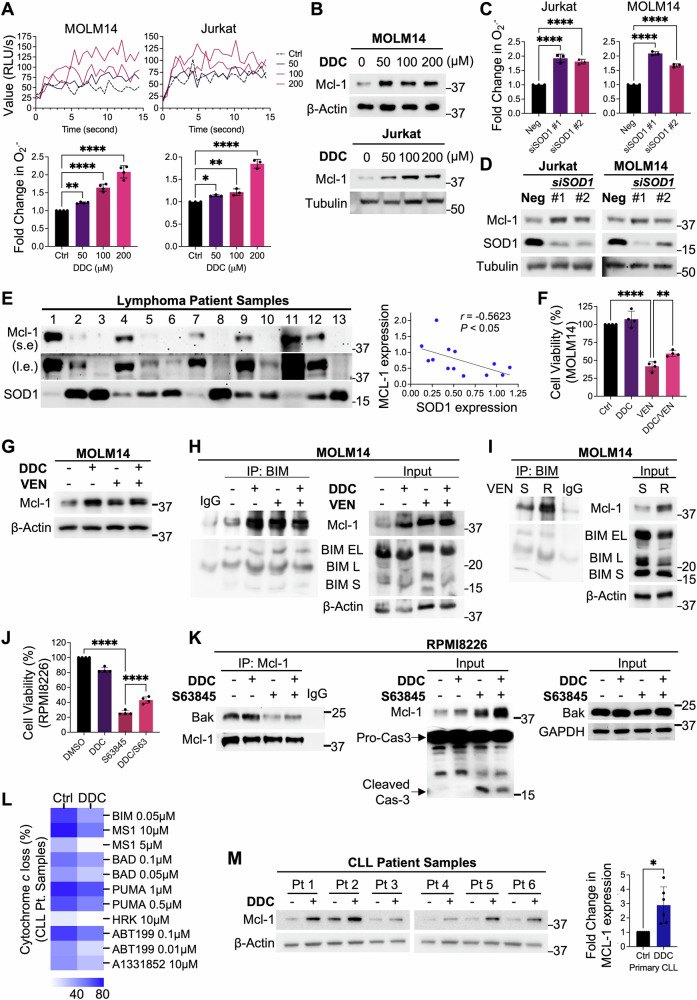
Increase in intracellular O_2_^.−^ induces Mcl-1 accumulation and apoptosis inhibition. **A** Line graph showing raw O_2_^.−^ detection following treatment with diethyldithiocarbamate (DDC) (50–200 µM) in MOLM14 or Jurkat cells for 4 h. Bar graphs directly below show fold change of averaged intracellular O_2_^.−^ levels upon treatment with indicated doses of DDC in MOLM14 (*N* = 4) or Jurkat (*N* = 3) cells for 4 h. Intracellular O_2_^.−^ level was measured via lucigenin chemiluminescence assay. Holm–Sidak’s comparisons test was used. **B** Western blot analyses showing increase in Mcl-1 levels upon treatment with indicated doses of diethyldithiocarbamate (DDC) in MOLM14 or Jurkat cells for 4 h. *N* = 3. **C** Graph showing fold change in averaged intracellular O_2_^.−^ levels upon treatment with *siRNA*-mediated knockdown of superoxide dismutase 1 (*SOD1*) (100 nM) in Jurkat (left panel) or MOLM14 (right panel) cells for 48 h. Intracellular O_2_^.−^ level was measured via lucigenin chemiluminescence assay. *N* = 3. Holm–Sidak’s comparisons test was used. **D** Western blot analyses showing Mcl-1, SOD1, and tubulin levels after *siRNA*-mediated knockdown of *SOD1* (100 nM) in Jurkat or MOLM14 cells for 48 h. *N* = 3. **E** Western blot showing Mcl-1 and SOD1 levels in 13 lymphoma patient samples. The top panel (s.e) represents short exposure of the blot whereas the panel below (l.e) represents a long exposure of the blot. Densitometric analysis and Pearson correlation analysis of Mcl-1 (l.e.) vs SOD1 protein levels were performed from 13 lymphoma patient samples. Raw densitometric values obtained from ImageJ were used for plotting the graph. *N* = 13. **F** Cell viability of MOLM14 cells treated with DDC (25 µM) for 2 h before treatment with VEN (0.02 μM) for 48 h, measured via MTT assay. *N* = 4. Holm–Sidak’s comparisons test was used. **G** Western blot showing Mcl-1 levels of MOLM14 cells following treatment with DDC (25 µM) for 2 h before treatment with VEN (0.02 μM) for 48 h. *N* = 3. **H** Co-immunoprecipitation of BIM isoforms and immunoblots of BIM isoforms and Mcl-1 in MOLM14 cells treated with DDC (25 µM) for 2 h before treatment with VEN (0.02 μM) for subsequent 24 h. Input showing levels of Mcl-1, BIM, β-Actin. *N* = 2. **I** Co-immunoprecipitation of BIM isoforms and immunoblots of BIM isoforms and Mcl-1 in VEN-S and VEN-R MOLM14 cells. Input showing levels of Mcl-1, BIM, β-Actin. *N* = 2. **J** Cell viability of RPMI8226 cells treated with DDC (100 µM) for 2 h before treatment with S63845 (0.075 μM) for 24 h, measured via MTT assay. *N* = 4. Holm–Sidak’s comparisons test was used. **K** Co-immunoprecipitation of Mcl-1 and immunoblots of Bak, and Mcl-1 in RPMI8226 cells treated with DDC (100 µM) for 2 h before treatment with S63845 (0.075 μM) for subsequent 48 h. Input showing levels of Mcl-1, pro and cleaved caspase-3, Bak and GAPDH. *N* = 3. **L** Heatmap of BH3-profiling showing percentage of cytochrome *c* loss from CLL patient samples after ex vivo treatment with DDC (50 µM) for 4 h and subsequent exposure to different BH3 peptide or mimetic concentrations. *N* = 6. BH3 Peptides—BIM or PUMA targets all pro-apoptotic proteins, MS1 targets Mcl-1, BAD targets Bcl-2 and Bcl-xL, HRK targets Bcl-xL, ABT199 targets Bcl-2, and A1331852 targets Bcl-xL. **M** Western blot showing Mcl-1 levels of CLL patient samples after ex vivo treatment with DDC (50 µM) for 4 h. *N* = 6. Densitometric analyses of Mcl-1/β-Actin levels in CLL patient samples treated with DDC (50 µM) for 4 h, normalized to non-treated control. *N* = 6. Paired *t*-test was used.

Next, we questioned if the intracellular increase in O_2_^.−^ was upstream of Mcl-1 accumulation. Intracellular O_2_^.−^ was manipulated via pharmacological inhibition (diethyldithiocarbamate; DDC) of superoxide dismutase-1 (SOD1), an enzyme responsible for the conversion of O_2_^.−^ to hydrogen peroxide (H_2_O_2_), or its genetic knockdown (*siSOD1*). DDC treatment resulted in a dose-dependent increase in intracellular O_2_^.−^ in VEN-S MOLM14 and OCI-AML2 AML cells and three other hematopoietic cell lines, RPMI8226 myeloma, Jurkat T-ALL, and OCI-Ly3 DLBCL (Figs. 2A and S3A). Importantly, increased intracellular O_2_^.−^ resulted in Mcl-1 accumulation in all 5 cell lines, indicating that the effect of O_2_^.−^ on Mcl-1 was not limited to AML cell lines (Figs. 2B and S3A). Similarly, *siSOD1* resulted in an increase in intracellular O_2_^.−^ and Mcl-1 protein levels (Figs. 2C, D and S3B). To further validate these in vitro data, we analyzed the expression of Mcl-1 and SOD1 in a panel of patient-derived lymphoma biopsies. Indeed, analysis of lysates from 13 lymphoma patients revealed a significant inverse correlation between Mcl-1 and SOD1 protein levels in vivo, validated by Pearson correlation analysis (Fig. 2E).

Intrigued by the association between O_2_^.−^ and Mcl-1 in VEN-R cells, we next evaluated if increase in O_2_^.−^ could affect sensitivity to VEN. Pre-treatment of VEN-S MOLM14 cells with DDC significantly reduced cell sensitivity to VEN (Fig. 2F), in conjunction with increased Mcl-1 expression (Fig. 2G). In support of the latter finding as well as our recent publication demonstrating that reduced binding of the pro-apoptotic executioner BIM to BCL-2, is concurrently sequestered by the increased Mcl-1 protein in VEN-R DLBCL cells [7], our immunoprecipitation assay revealed that increased Mcl-1 could further scavenge BIM in VEN-S MOLM14 cells (Fig. 2H), thus suggesting that the increased Mcl-1 protein is phenotypically functional through the reduction of apoptosis. Importantly, the protein-protein interactive effect between stabilized Mcl-1 and BIM was also recapitulated in the VEN-R MOLM14 cells (Fig. 2I). These data provide testimony that O_2_^.−^-mediated decrease in VEN sensitivity is a function of active Mcl-1 protein. To further confirm that, we assessed the effect of increased O_2_^.−^ on cell death upon exposure to Mcl-1 specific inhibitor, S63845. Similar to results observed with VEN, incubation of S63845-sensitive RPMI8226 cells with DDC resulted in a significant reduction in cell death (Fig. 2J). Furthermore, immunoprecipitation analysis revealed that S63845-mediated dissociation of Mcl-1 from Bak was reversed upon DDC-induced increase in O_2_^.−^, corresponding to an increased Mcl-1 level and decreased cleaved caspase 3 (Fig. 2K). These data imply that increased Bak and/or BIM sequestration by Mcl-1 could reduce apoptotic sensitivity by interfering with MOMP in cancer cells harboring elevated O_2_^.−^ levels. Indeed, annexin V assay confirms that DDC could rescue VEN- and S63845-induced apoptotic cell death in VEN-S MOLM14 cells (Fig. S4A). To further corroborate our findings, we employed BH3-profiling to evaluate whether elevated intracellular O_2_^.−^ affects mitochondria priming in CLL patient samples. Patient-derived CLL cells were treated ex vivo with DDC and cytochrome *c* loss was measured upon incubation with various BH3-peptides/mimetics. Results showed decreased overall apoptotic priming (reduced cytochrome *c* loss for all peptides) in DDC-treated patient-derived CLL cells (Fig. 2L). Importantly, reduced priming was also associated with a significant increase in Mcl-1 levels in the 6 patient-derived CLL cells treated ex vivo with DDC (Fig. 2M). Together, these findings establish the role of aberrant O_2_^.−^ signaling on Mcl-1 upregulation, which regulates MOMP by sequestering pro-apoptotic proteins to render cells resistant to VEN-induced apoptosis.

### Reduction in intracellular O_2_^.−^ levels decreases Mcl-1 and restores venetoclax sensitivity

**Fig. 3:**
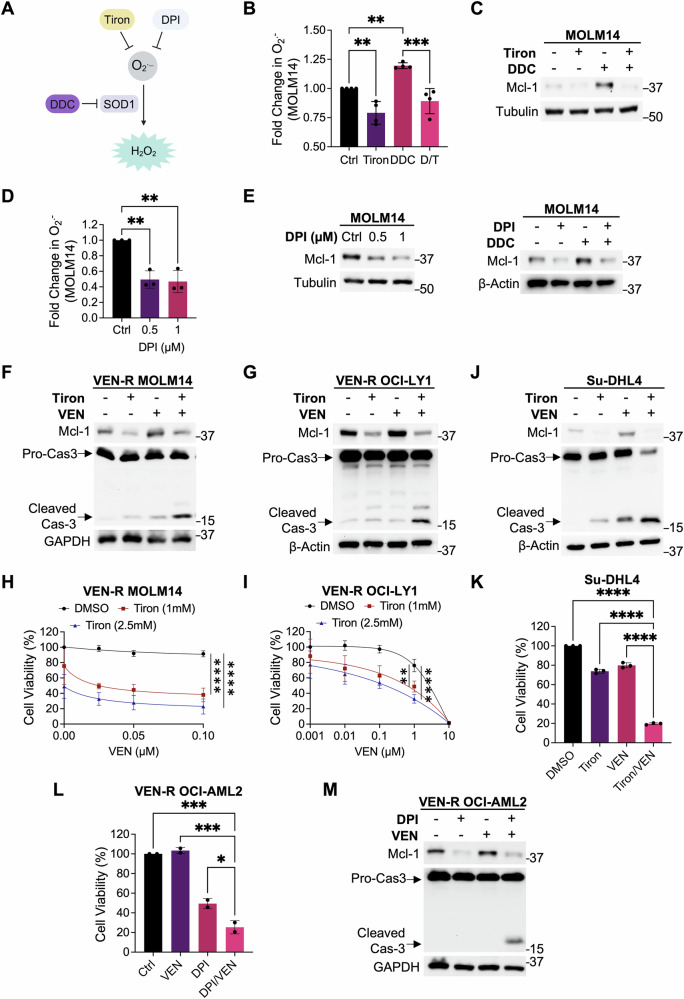
Scavenging intracellular O_2_^.−^ reduces Mcl-1 level and increases apoptotic sensitivity. **A** Diagram illustrating the conversion of superoxide (O_2_^.−^) to hydrogen peroxide (H_2_O_2_) is mediated by superoxide dismutase 1 (SOD1), which could be inhibited by diethyldithiocarbamate (DDC). Tiron and DPI are scavengers and inhibitors of O_2_^.−^ production, respectively. The diagram was created in BioRender. Pervaiz, S. (2022) BioRender.com/v93n781. **B** Graph showing fold change in intracellular O_2_^.−^ levels in MOLM14 cells upon treatment with Tiron (5 mM) for 1 h followed by DDC (50 µM) for 4 h. *N* = 4. Holm–Sidak’s comparisons test was used. **C** Western blot showing Mcl-1 levels in MOLM14 cells treated with O_2_^.−^ scavenger, Tiron (5 mM) for 1 h followed by DDC (50 µM) for 4 h. *N* = 3. **D** Graph showing fold change of intracellular O_2_^.−^ levels in MOLM14 cells upon treatment with DPI for 20 h. Intracellular O_2_^.−^ levels was measured via lucigenin chemiluminescence assay. *N* = 3. Dunnett’s multiple comparisons test was used. **E** Western blot showing Mcl-1 levels in MOLM14 cells upon treatment with increasing diphenyleneiodonium (DPI) doses for 20 h (left panel) or pre-treatment with DPI (1 μM) for 20 h and in combination with DDC (50 μM) for subsequent 4 h (right panel). *N* = 3. **F**, **G** Western blot showing Mcl-1, pro and cleaved caspase-3, GAPDH and/or β-Actin levels of VEN-R MOLM14 or VEN-R OCI-Ly1 cells following treatment with O_2_^.−^ scavenger, Tiron (1 mM/VEN-R MOLM14, 2.5 mM/VEN-R OCI-Ly1) for 2 h followed by VEN (0.05 µM/VEN-R MOLM14, 1 µM/VEN-R OCI-Ly1) for 48 h. *N* = 3. **H**, **I** Cell viability of VEN-R MOLM14 (*N* = 4) or VEN-R OCI-Ly1 (*N* = 4) cells following treatment with O_2_^.−^ scavenger, Tiron, for 2 h followed by increasing VEN doses for 48 h, measured via CTG assay. Tukey’s multiple comparisons test was used. **J** Western blot showing Mcl-1, pro and cleaved caspase-3, GAPDH and/or β-Actin levels of inherently VEN-R Su-DHL4 cells following treatment with O_2_^.−^ scavenger, Tiron (5 mM) for 2 h followed by VEN (0.5 µM) for 48 h. *N* = 3. **K** Cell viability of inherently VEN-R Su-DHL4 cells following treatment with O_2_^.−^ scavenger, Tiron (5 mM) for 2 h followed by VEN (0.5 µM) for 48 h, measured via CTG assay. *N* = 3. Sidak’s multiple comparisons test was used. **L** Cell viability of acquired VEN-R OCI-AML2 cells following treatment with DPI (0.1 µM) for 2 h followed by VEN (0.2 µM) for 24 h. Cell viability was measured via MTT assay. *N* = 2. Sidak’s multiple comparisons test was used. **M** Western blot showing Mcl-1, pro and cleaved caspase-3 and GAPDH levels of VEN-R OCI-AML2 cells treated with DPI (0.1 µM) for 2 h followed by VEN (0.2 µM) for 24 h. *N* = 3.

To validate that increased Mcl-1 is a function of increased O_2_^.−^, we employed two different strategies to reduce intracellular O_2_^.− ^– scavenging O_2_^.−^ with Tiron or inhibiting NADPH oxidase(NOX)-dependent O_2_^.−^ production with diphenyleneiodonium (DPI) (Fig. 3A). Tiron pre-treatment neutralized DDC-induced increases in intracellular O_2_^.−^ and Mcl-1 expression in VEN-S MOLM14 cells (Fig. 3B, C). Similarly, DPI treatment reduced intracellular O_2_^.−^ levels as well as baseline and DDC-induced Mcl-1 expression in VEN-S MOLM14 cells (Fig. 3D, E), thus confirming that increased Mcl-1 protein is attributed to increased intracellular O_2_^.−^.

To further evaluate the effects of O_2_^.−^ on Mcl-1 specifically in VEN-R cells, we proceeded to treat VEN-R MOLM14 cells with VEN at increasing concentrations. We observed a concomitant increase in O_2_^.−^ and Mcl-1, suggesting a similar regulation of O_2_^.−^ on Mcl-1 in VEN-R cells (Fig. S5A, B). To confirm that O_2_^.−^ is upstream of Mcl-1 stability, we treated VEN-R cells with the O_2_^.−^ scavenger, Tiron, and successfully reduced not only baseline Mcl-1 but also VEN-induced Mcl-1 in VEN-R MOLM14 and VEN-R OCI-Ly1 cells, thus confirming that O_2_^.−^ is indeed an upstream regulator of Mcl-1 stability (Fig. 3F, G). Importantly, reduced intracellular O_2_^.−^ by Tiron was further translated to an increased caspase-3 cleavage and decrease in cell viability upon co-treatment with VEN in VEN-R MOLM14 and VEN-R OCI-Ly1 cells, as measured by Western blot analysis, CellTiter-Glo (CTG), and Annexin V cell viability assays (Figs. 3F–I and S6A). Consistent effects of scavenging O_2_^.−^ were also observed in inherently VEN-R Su-DHL4 [7] and OCI-AML3 cells (Figs. 3J, K and S6B, C). To further corroborate these findings, we assessed the effect of inhibiting NOX-dependent O_2_^.−^ generation (DPI) on VEN sensitivity in VEN-R OCI-AML2 cells. DPI treatment similarly reduced cell viability, Mcl-1 levels and increase caspase-3 cleavage in VEN-R OCI-AML2 cells upon co-treatment with VEN (Fig. 3L, M). Finally, it is worth pointing out that Tiron or DPI alone significantly reduced Mcl-1 levels, thus strongly supporting the involvement of intracellular O_2_^.−^ on Mcl-1 protein levels. Together, these data indicate that modulating intracellular O_2_^.−^ reduces Mcl-1 levels and restores sensitivity of VEN-R cells to VEN.

### Increased O_2_^.−^ induces Mcl-1 abundance by reducing its protein turnover

**Fig. 4:**
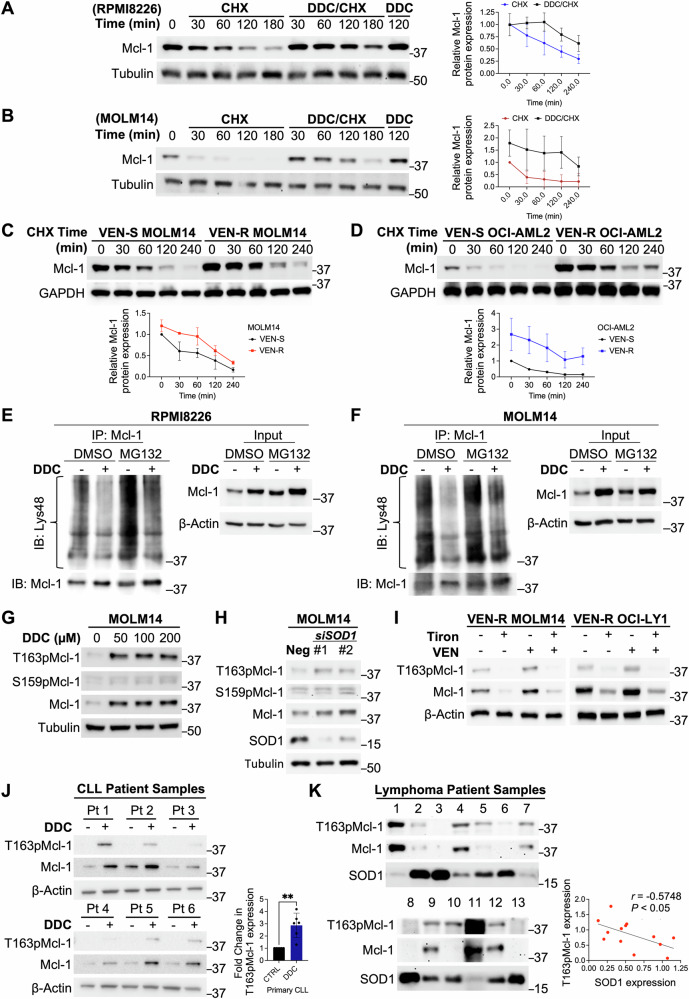
O_2_^.−^ induces Mcl-1 stability through post-translational modification of phosphorylation. Western blot showing Mcl-1 and tubulin levels in **A** RPMI8226, **B** MOLM14 cells following pre-treatment with DDC (100 µM) for 120 min followed by cycloheximide (CHX) 25 µg/ml, for the indicated timepoints (in minutes). Right panels: Graph plotting the densitometric analysis of RPMI8226 or MOLM14 cells treated with DDC and CHX or CHX alone. Fold change in densitometry values (Mcl-1/Tubulin) and were normalized to untreated control at 0 min of the experiment. Densitometric values were obtained using ImageJ. *N* = 3. **C**, **D** Western blot showing Mcl-1 and GAPDH levels in MOLM14, VEN-R MOLM14, OCI-AML2, VEN-R OCI-AML2 following treatment of cycloheximide (CHX) (25 µg/ml) for the indicated time points. Bottom panels: Graph plotting the densitometric analysis of MOLM14, VEN-R MOLM14, OCI-AML2, and VEN-R OCI-AML2 cells. Densitometric values were obtained using ImageJ. *N* = 3. **E**, **F** Co-Immunoprecipitation of Mcl-1 and immunoblots of Lys48 (K48) and Mcl-1 in RPMI8226 or MOLM14 cells pre-treated with MG132 (5 µM) for 1 h, followed by DDC (100 µM) for 4 h. Input showing levels of Mcl-1 and β-Actin. *N* = 2. **G** Western blot showing T163pMcl-1, S159pMcl-1, Mcl-1, Tubulin levels in MOLM14 cells after treatment with indicated doses of DDC (µM) for 4 h. *N* = 3. **H** Western blot showing T163pMcl-1, S159pMcl-1, Mcl-1, SOD1, Tubulin levels in MOLM14 cells after *siRNA*-mediated knockdown of *SOD1* (100 nM) for 48 h. *N* = 3. **I** Western blot showing T163pMcl-1, Mcl-1, β-Actin levels in VEN-R MOLM14 or VEN-R OCI-Ly1 following treatment with O_2_^.−^ scavenger, Tiron (1 mM/VEN-R MOLM14, 2.5 mM/VEN-R OCI-Ly1) for 2 h followed by VEN (0.05μM/VEN-R MOLM14, 1μM/VEN-R OCI-Ly1) for 48 h. *N* = 3. **J** Western blot showing T163pMcl-1, Mcl-1, β-Actin levels of CLL patient samples after ex vivo treatment with DDC (50 µM) for 4 h. Densitometric analyses of T163pMcl-1/β-Actin levels in CLL patient samples treated with DDC (50 µM) for 4 h, normalized to non-treated control. *N* = 6. Mcl-1 and β-Actin are from the same set in Fig. 2M. Paired *t*-test was used. **K** Western blot showing T163pMcl-1, Mcl-1, and SOD1 levels in 13 lymphoma patient samples. Densitometric analysis and Pearson correlation analysis of T163pMcl-1 vs SOD1 protein levels was performed from 13 lymphoma patient samples. Raw densitometric values obtained from ImageJ were used for plotting the graph. Mcl-1 and SOD1 are from the same set in Fig. 2E. *N* = 13.

We next investigated the underlying mechanism of O_2_^.−^-mediated Mcl-1 accumulation. As Mcl-1 could be regulated through gene transcription or protein stability, we first investigated whether increased O_2_^.−^ could affect gene transcription. Real-time PCR analysis of *MCL1* mRNA following DDC treatment revealed no significant difference in *MCL1* mRNA levels in VEN-S MOLM14 or RPMI8226 cells (Fig. S7A, B), thus indicating that the effect of increased intracellular O_2_^.−^ on Mcl-1 could be downstream of gene transcription. We next performed a cycloheximide (CHX) time-chase experiment to determine if the effect was mediated at the post-translational level. Results showed that pre-treatment with DDC for 2 h followed by CHX for the indicated timepoints clearly increased the half-life of Mcl-1 protein in RPMI8226 or VEN-S MOLM14 cells (Fig. 4A, B). Notably, similar results of longer Mcl-1 half-life were observed in VEN-R MOLM14 and VEN-R OCI-AML2 cells, thus corroborating our findings in the context of VEN resistance (Fig. 4C, D). These data indicate that Mcl-1 is post-translationally stabilized in VEN-R cells in a milieu of heightened intracellular O_2_^.−^ levels.

Since O_2_^.−^-mediated Mcl-1 stability occurs post-translationally, we investigated if this was due to a reduction in protein turnover. As Mcl-1 protein stability is regulated by the proteasomal degradation pathway [20], we inhibited proteasomal degradation pathway with the proteasomal inhibitor, MG132, to accumulate Mcl-1 and subsequently treated cells with DDC. We hypothesized that MG132-accumulated Mcl-1 that are stabilized by DDC-induced O_2_^.−^ would display lower Lys48 (K48) polyubiquitin chain modification, a key signal for protein degradation via the proteasome [20, 36], as compared to MG132-accumulated Mcl-1 without DDC. Indeed, we observed vividly lower levels of Lys48 polyubiquitinated-Mcl-1 in cells that were treated with DDC (Fig. 4E). Importantly, lower Lys48 polyubiquitination corresponded accurately with higher levels of Mcl-1 in our input (Fig. 4E). These results were consistently observed in two different cell lines of RPMI8226 and VEN-S MOLM14 cells (Fig. 4E, F), thereby indicating that increase in intracellular O_2_^.−^ promotes Mcl-1 protein stabilization and accumulation by inhibiting its proteasomal degradation.

### O_2_^.−^-mediated Mcl-1 stability is a function of its Thr163 phosphorylation

Mcl-1 stability is regulated by its phosphorylation at Thr163 (T163pMcl-1) and/or S159 (S159pMcl-1), whereby T163pMcl-1 promotes its stability while S159pMcl-1 promotes its proteasomal degradation [22, 25]. Since we observed a prolonged half-life of Mcl-1 and reduced ubiquitination-mediated degradation of Mcl-1, we postulated that Mcl-1 phosphorylation is affected by the altered O_2_^.−^ levels. Indeed, significant increases in the stabilizing T163pMcl-1 and Mcl-1, but not the degrading S159pMcl-1, were detected upon DDC-induced O_2_^.−^ elevation in VEN-S MOLM14 cells (Fig. 4G). Similar results were obtained upon knocking down *SOD1* with *siSOD1* (Fig. 4H). Reciprocally, scavenging O_2_^.−^ with Tiron in VEN-R MOLM14, OCI-Ly1 or Su-DHL4 cells resulted in reduced T163pMcl-1 levels (Figs. 4I and S8A). We further recapitulated these in vitro findings in ex vivo DDC-treated CLL primary cells by observing that DDC could elevate T163pMcl-1 (Fig. 4J). Finally, in an in vivo setting, we observed a significant inverse correlation between T163pMcl-1 and SOD1 protein expression levels in a range of 13 lymphoma patient samples (Fig. 4K). These data provide evidence that increased O_2_^.−^ stabilizes Mcl-1 by promoting T163 phosphorylation.

**Fig. 5:**
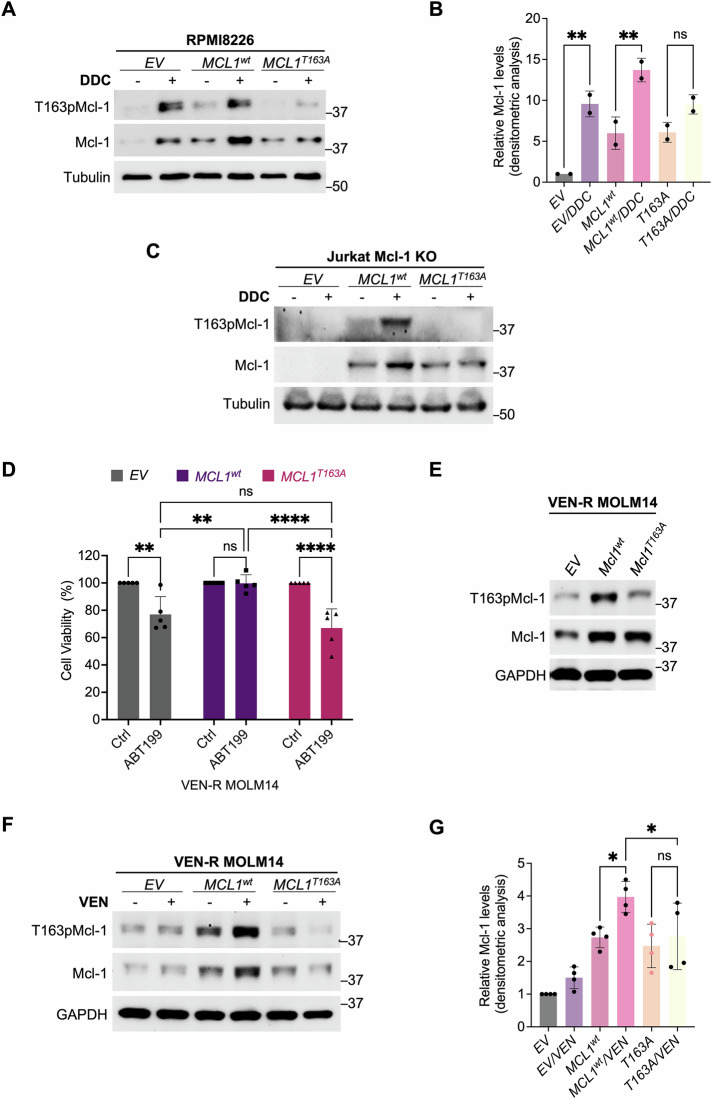
O_2_^.−^-dependent increase in Mcl-1 stability is a function of T163pMcl-1. **A** Western blot showing T163pMcl-1, Mcl-1, Tubulin levels in RPMI8226 cells after transfection with 2 µg of empty vector (*EV*), *MCL1*^*wt*^, or non-phosphorylatable *MCL1*^*T163A*^ for 48 h followed by treatment with DDC (100 µM) for 4 h. *N* = 2. **B** Densitometric analyses of Mcl-1 levels in RPMI8226 cells after transfection with 2 µg of empty vector (*EV*), *MCL1*^*wt*^, or non-phosphorylatable *MCL1*^*T163A*^ for 48 h followed by treatment with DDC (100 µM) for 4 h. Densitometric values were normalized to tubulin (Mcl-1/Tubulin) and results are relative to *EV*. *N* = 2. Sidak’s multiple comparisons test was used. **C** Western blot showing T163pMcl-1, Mcl-1 levels in Jurkat Mcl-1 KO cells after transfection with 2 µg of empty vector (*EV*), *MCL1*^*wt*^, or non-phosphorylatable *MCL1*^*T163A*^ for 48 h followed by treatment with DDC (100 µM) for 4 h. *N* = 3. **D** Graph showing cell viability of VEN-R MOLM14 cells transfected with 2 µg of empty vector (*EV*), *MCL1*^*wt*^, or non-phosphorylatable *MCL1*^*T163A*^ for 24 h followed by treatment with VEN (0.05 μM) for 48 h. Cell viability was measured using MTT assay. *N* = 5. Tukey’s multiple comparisons test was used. **E** Western blot showing T163pMcl-1, Mcl-1, and GAPDH of VEN-R MOLM14 cells transfected with 2 µg of empty vector (*EV*), *MCL1*^*wt*^, or non-phosphorylatable *MCL1*^*T163A*^ for 24 h. *N* = 3. **F** Western blot showing T163pMcl-1, Mcl-1, and GAPDH of VEN-R MOLM14 cells transfected with 2 µg of empty vector (*EV*), *MCL1*^*wt*^, or non-phosphorylatable *MCL1*^*T163A*^ for 24 h followed by treatment with VEN (0.05 μM) for 24 h. *N* = 4. **G** Densitometric analyses of Mcl-1 levels in VEN-R MOLM14 cells transfected with 2 µg of empty vector (*EV)*, *MCL1*^*wt*^, or non-phosphorylatable *MCL1*^*T163A*^ for 24 h followed by treatment with VEN (0.05 μM) for 24 h. Densitometric values were normalized to GAPDH (Mcl-1/GAPDH), and results are relative to *EV*. N = 4. Sidak’s multiple comparisons test was used.

To validate the role of T163pMcl-1 in promoting Mcl-1 stability upon increased intracellular O_2_^.−^, we transfected cells with empty vector (*EV*), wild-type *MCL1* (*MCL1*^*wt*^), or phospho-deficient mutant-T163A *MCL1* (*MCL1*^*T163A*^), followed by DDC treatment. Notably, the DDC-induced increase in Mcl-1 was observed in cells overexpressing *MCL1*^*wt*^, but not upon overexpression of *MCL1*^*T163A*^ (Fig. 5A, B). Subsequently, we generated a stable *CRISPR/Cas9* Mcl-1 knockout (KO) clone of Jurkat cells. Note that VEN-R MOLM14 cells were not used due to their survival dependence on Mcl-1 (Fig. S9A). These cells were not suitable for the generation of a stable *CRISPR/Cas9* Mcl-1 KO clone due to the potential significant cell death or adaptive mechanisms that may hinder subsequent investigation pertaining to Mcl-1 and its dependence. The Jurkat Mcl-1 KO cells were subsequently transiently transfected with *EV, MCL1*^*wt*^*, or MCL1*^*T163A*^ and treated with DDC. Notably, while DDC-induced O_2_^.−^ increase resulted in Mcl-1 upregulation in *MCL1*^*wt*^*-*expressing cells, there was no observable increase upon transfection with *MCL1*^*T163A*^ (Fig. 5C). Together, these data provide evidence to link O_2_^.-^-mediated Mcl-1 stability to T163pMcl-1.

Next, we verified if T163pMcl-1 was involved in regulating Mcl-1 stability in the context of VEN resistance. VEN-R MOLM14 cells were transfected with *EV*, *MCL1*^*wt*^ or *MCL1*^*T163A*^ for 24 h followed by treatment with VEN for 48 h (Fig. 5D, E). Notably, *MCL1*^*T163A*^-expressing cells were significantly more sensitive to VEN compared to *MCL1*^*wt*^-expressing cells (Fig. 5D). Furthermore, unlike *MCL1*^*wt*^-expressing cells, VEN treatment failed to upregulate Mcl-1 in *MCL1*^*T163A*^-expressing cells (Fig. 5F, G). Collectively, these data indicate that enhanced Mcl-1 stability induced by increased O_2_^.−^ is dependent on T163pMcl-1, which appears to be critical in the context of acquired VEN resistance.

### AKT activation underlies O_2_^.−^-induced T163pMcl-1 and Mcl-1 stability

**Fig. 6:**
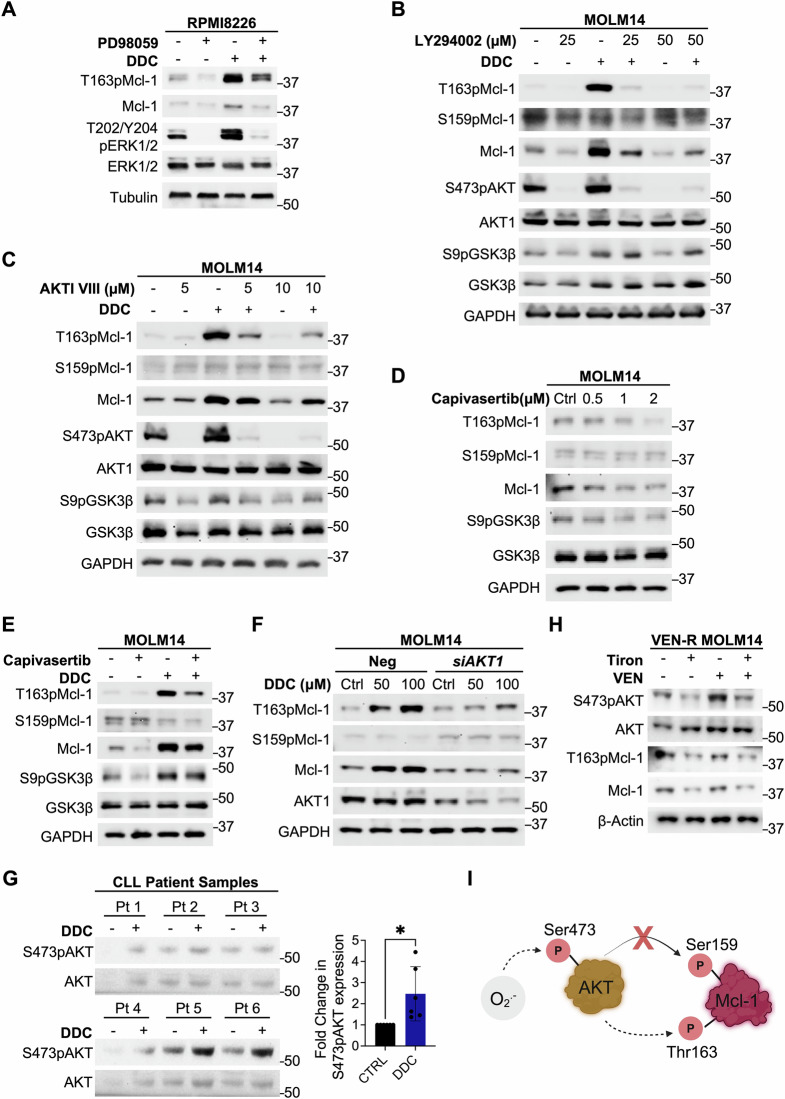
AKT activation underlies O_2_^.−^-induced Mcl-1 stability. **A** Western blot showing T163pMcl-1, Mcl-1, T202/Y204pERK, ERK1/2, Tubulin levels of RPMI8226 cells pre-treated with PD98059 (10 µM) followed by DDC (100 µM) for 4 h. *N* = 3. **B** Western blot showing T163pMcl-1, S159pMcl-1, Mcl-1, S473pAKT, AKT1, S9pGSK3β, GSK3β, GAPDH levels of MOLM14 cells pre-treated with LY294002 (25/50 µM) for 2 h followed by DDC (100 µM) for 4 h. *N* = 3. **C** Western blot showing T163pMcl-1, S159pMcl-1, Mcl-1, S473pAKT, AKT1, S9pGSK3β, GSK3β, GAPDH levels of MOLM14 cells pretreated with AKT inhibitor VIII (25/50 µM) for 2 h followed by DDC (100 µM) for 4 h. *N* = 3. **D** Western blot showing T163pMcl-1, S159pMcl-1, Mcl-1, S9pGSK3β, GSK3β, GAPDH levels of MOLM14 cells treated with indicated doses of capivasertib (µM) for 5 h. *N* = 3. **E** Western blot showing T163pMcl-1, S159pMcl-1, Mcl-1, S9pGSK3β, GSK3β, GAPDH levels of MOLM14 cells pre-treated with capivasertib (1 µM) for 1 h and followed by DDC (100 µM) for 4 h. *N* = 3. **F** Western blot showing T163pMcl-1, S159pMcl-1, Mcl-1, AKT1, GAPDH levels of MOLM14 cells transfected with Neg or *siAKT1* for 48 h before treatment with DDC (50–100 µM) for 4 h *N* = 3. **G** Western blot showing S473pAKT, AKT levels of CLL patient samples after ex vivo treatment with DDC (50 µM) for 4 h. Densitometric analyses of S473pAKT/AKT levels in CLL patient samples treated with DDC (50 µM) for 4 h, normalized to non-treated control. *N* = 6. Paired *t-*test was used. **H** Western blot showing S473pAKT, AKT, T163pMcl-1, Mcl-1, β-Actin levels in VEN-R MOLM14 following treatment with O_2_^.−^ scavenger, Tiron (1 mM) for 2 h before treatment with VEN (0.05 μM) for 48 h. *N* = 3. **I** Diagram illustrating that O_2_^.−^-induced T163pMcl-1, but not S159pMcl-1, is dependent on AKT activation. The diagram was Created in BioRender. Pervaiz, S. (2022) BioRender.com/p32o555.

T163pMcl-1 has been linked to ERK activation [24] and we observed that ERK inhibition via PD98059 blocked DDC-induced Mcl-1 upregulation, thus indicating the involvement of ERK in promoting O_2_^.−^-mediated Mcl-1 stability (Fig. 6A). Interestingly, earlier reports provide evidence that AKT could physically interact with Mcl-1 [37] as well as its enzymatic activity is activated by O_2_^.−^ [38]. Given these observations, we questioned the role of AKT in mediating redox-dependent T163pMcl-1 and Mcl-1 stability. First, DDC treatment resulted in AKT activation, evidenced by increased S473 phosphorylation (S473pAKT) and downstream GSK3β S9 phosphorylation (S9pGSK3β), as well as increased T163pMcl-1 and Mcl-1 (Fig. 6B). Second, pre-treatment with PI3K inhibitor, LY294002, prevented DDC-induced AKT activation and blocked T163pMcl-1 and Mcl-1 accumulation (Fig. 6B). Third, AKT-specific inhibitor-VIII (AKTI-VIII) prevented DDC-induced increase in S473pAKT, and importantly, inhibited the increase in T163pMcl-1 and Mcl-1 (Fig. 6C). Intriguingly, although blocking PI3K or AKT reduced inhibitory S9pGSK3β, there was no significant increase in GSK3β-dependent S159pMcl-1, which promotes Mcl-1 degradation (Fig. 6B, C). Fourth, we used capivasertib, an AKT inhibitor currently approved for breast cancer treatment [39], and observed that capivasertib could significantly reduce T163pMcl-1 and Mcl-1 levels without affecting S159pMcl-1 (Fig. 6D). As capivasertib does not prevent S473pAKT but competitively binds to its ATP-binding pocket to inhibit downstream phosphorylation of its target [40, 41], we therefore used S9pGSK3β as a positive control (Fig. 6D). Furthermore, pre-treatment with capivasertib for 1 h prevented O_2_^.−^-mediated T163pMcl-1 and Mcl-1 accumulation by DDC (Fig. 6E). Similarly, *siAKT1* could prevent DDC-induced increases in T163pMcl-1 and Mcl-1 levels in MOLM14 and OCI-AML3 cells (Figs. 6F and S10A). It is worth pointing out that the link between AKT activation and O_2_^.−^-mediated Mcl-1 accumulation is not exclusive to hematopoietic cancers; stable knockout of *AKT1/2* in HCT116 colorectal carcinoma cells (*AKT-DKO*) also exhibited reduced T163pMcl-1 and Mcl-1 levels, which was not due to reduction in *MCL1* mRNA levels (Fig. S10B, C). Importantly, ex vivo DDC-treated primary CLL patient samples showed a significant increase in S473pAKT (Fig. 6G), in conjunction with downstream increase in T163pMcl-1 and Mcl-1 (Fig. 4J). Further validating the involvement of O_2_^.−^ in regulating S473pAKT and T163pMcl-1 in the context of VEN resistance, results show that scavenging O_2_^.−^ with Tiron reduced S473pAKT, T163pMcl-1, and Mcl-1 in VEN-R MOLM14 cells (Fig. 6H). Together, these data highlight the importance of AKT in redox-dependent stability of Mcl-1 via T163pMcl-1, particularly in VEN-R cells (Fig. 6I).

### Targeting hyperactivated AKT in venetoclax-resistant cells restores sensitivity to venetoclax

**Fig. 7:**
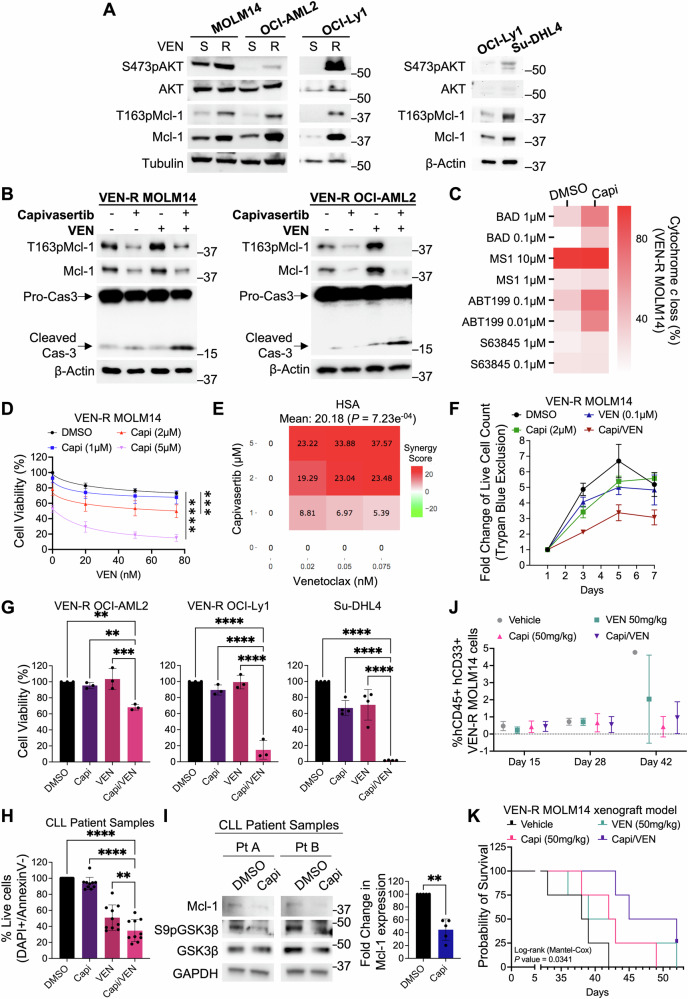
Inhibition of AKT restores sensitivity of venetoclax-resistant cells to venetoclax. **A** Western blot showing S473pAKT, AKT, T163pMcl-1, Mcl-1, Tubulin and/or β-Actin levels of VEN-S and VEN-R MOLM14, OCI-AML2, and OCI-Ly1 cells, as well as inherently VEN-R Su-DHL4 cells. **B** Western blot showing T163pMcl-1, Mcl-1, pro and cleaved caspase-3, β-Actin levels in VEN-R MOLM14 or VEN-R OCI-AML2 cells treated with capivasertib (1 µM/VEN-R MOLM14, 5 µM/VEN-R OCI-AML2) and/or VEN (0.1 µM/VEN-R MOLM14, 10 µM/VEN-R OCI-AML2) for 24 h. *N* = 3. **C** Heatmap of BH3-profiling showing percentage of cytochrome *c* loss from VEN-R MOLM14 cells following treatment with capivasertib (2 µM) or DMSO for 4 h, and subsequent exposure to different BH3 peptide or mimetic concentrations to indicate specific increase in Bcl-2 dependence. *N* = 3. **D** Graph showing cell viability assay of VEN-R MOLM14 cells treated with capivasertib (1–5 µM) and/or VEN (0.02–0.075 μM) for 48 h. Cell viability was assessed using CTG assay and data were normalized to untreated control cells. *N* = 3. Sidak’s multiple comparisons test was used. **E** Heatmap below showing synergy score based on Highest Single Agent (HSA) synergy model, calculated using SynergyFinder. **F** Graph showing a 7-day chase assay of VEN-R MOLM14 live cell count in fold change following treatment with DMSO, capivasertib (2 μM), VEN (0.1 μM), or combination. Day 3—DMSO vs combo *P* < 0.0001, Capi vs combo *P* < 0.02, VEN vs combo *P* < 0.001; Day 5—DMSO vs combo *P* < 0.0001, Capi vs combo *P* < 0.001, VEN vs combo *P* < 0.002; Day 7—DMSO vs combo *P* < 0.0001, Capi vs combo *P* < 0.0001, VEN vs combo *P* < 0.001. Tukey’s multiple comparisons test was used. **G** Cell viability assay of VEN-R OCI-AML2 (*N* = 3), VEN-R OCI-Ly1 (*N* = 3), Su-DHL4 (*N* = 4) cells treated with capivasertib and/or VEN for 48 h. Cell viability was assessed using the CTG assay and data were normalized to untreated control cells. Concentrations used for capivasertib (5 µM/VEN-R OCI-AML2, 0.5 µM/OCI-Ly1 VEN-R, 2 µM/Su-DHL4) and/or VEN (10 µM/VEN-R OCI-AML2, 1 µM/OCI-Ly1 VEN-R, 0.5 µM/Su-DHL4). Sidak’s multiple comparisons test was used. **H** Cell viability of CLL patient cells following ex vivo treatment with capivasertib (2 µM) and/or VEN (0.005 μM) for 24 h, measured via DAPI^+^/Annexin V^−^. *N* = 10. Sidak’s multiple comparisons test was used. **I** Western blot showing Mcl-1, S9pGSK3β﻿, GSK3β﻿, GAPDH levels in CLL patient cells following ex vivo treatment with capivasertib (2 µM) for 6 h. Representative blots shown for 2 CLL patient samples. Densitometric analyses of Mcl-1/β-Actin levels in CLL patient samples treated with capivasertib (2 µM) for 6 h, normalized to DMSO control. *N* = 5. Paired *t*-test was used. **J** Tumor burden of mice displayed by %hCD45 + hCD33 + VEN-R MOLM14 cells in blood sample harvested from mice that has been treated in vivo with vehicle, capivasertib, VEN or combo. *N* = 4 per study arm. Day 15 is the treatment initiation day. **K** Probability of survival following treatment with vehicle, capivasertib, VEN or combo in mice harboring VEN-R MOLM14 cells. *N* = 4 per study arm.

Given that VEN-R cells exhibit heightened O_2_^.−^, and AKT is activated by O_2_^.−^, we next questioned if AKT is hyperactivated in VEN-R cells. If so, would treatment with AKT inhibitors re-sensitize VEN-R cells to VEN? Indeed, S473pAKT levels (together with T163pMcl-1 and Mcl-1) were significantly higher in VEN-R MOLM14 and VEN-R OCI-AML2 (Fig. 7A). Simultaneously, T308pAKT was higher in VEN-R MOLM14 cells (Fig. S11A). Interestingly, VEN-R OCI-Ly1 and, inherently VEN-R Su-DHL4 also displayed higher S473pAKT levels (Fig. 7A). Since S473pAKT is upregulated in all VEN-R cells, we subsequently evaluated the effects of AKT inhibition on Mcl-1 levels and VEN sensitivity. Indeed, capivasertib not only reduced T163pMcl-1 and Mcl-1, but also enhanced caspase-3 cleavage upon VEN treatment in VEN-R MOLM14 and VEN-R OCI-AML2 cells (Fig. 7B). Additionally, BH3-profiling revealed that cells became more dependent on Bcl-2, thus more sensitive to Bcl-2 inhibition upon treatment with capivasertib (increased cytochrome *c* loss by BAD peptide or ABT199), which contrasted with MS1 peptide or S63845 where no further cytochrome *c* loss was induced (Fig. 7C). The increase in Bcl-2 dependence by capivasertib resulted in a synergistic reduction in cell viability of VEN-R MOLM14 cells when co-treated with VEN (Fig. 7D, highest single agent (HSA) synergy model, Figs. 7E [42, 43] and S12A). A 7-day cell proliferation chase assay via Trypan Blue exclusion further accentuated the cytotoxic effect of this treatment combination in significantly suppressing proliferation of VEN-R MOLM14 cells as compared to all control groups (Fig. 7F). The effects of this treatment combination were also recapitulated in other VEN-R cell lines of OCI-AML2, OCI-Ly1, and Su-DHL4 (Figs. 7B, G and S12A, B) and primary CLL patient samples (Figs. 7H, I and S13A). Interestingly, capivasertib did not drastically change the levels of S70pBcl-2 as compared to T163pMcl-1 and Mcl-1 in VEN-R MOLM14 and VEN-R OCI-AML2 cells (Figs. S14A and 7B), thus suggesting that the reduction in Mcl-1 alone is sufficient to re-sensitize VEN-R cells to VEN treatment. This indicates that Mcl-1 may play a more prominent role in VEN resistance, at least in myeloid malignancy.

Lastly, to verify our in vitro and ex vivo findings, we implanted VEN-R MOLM14 cells in NRG-SGM3 mice via tail vein and treated these mice according to our treatment schedule (Fig. S15A). Indeed, we observed that the treatment combination (capivasertib + VEN) not only reduced VEN-R MOLM14 cells systemically but also prolonged the survival of mice compared to those of the control groups (Fig. 7J, K). Importantly, all mice from the control groups reached the endpoint, but not those from the treatment combination group (Fig. 7K). Collectively, our finding demonstrates the interplay between AKT and Mcl-1 in driving VEN resistance and strongly suggest that AKT inhibition could be a potential therapeutic strategy to manage VEN-R cells and restore VEN sensitivity.

## Discussion

The use of VEN for clinical management of CLL and AML is a key milestone since the discovery of BH3-mimetic Bcl-2 inhibitor(s); however, relapse occurs due to the upregulation of a different anti-apoptotic protein Mcl-1 and switch in survival dependence. The upstream mechanism(s) underlying these changes upon VEN resistance acquisition still remain elusive. We present evidence linking intracellular O_2_^.−^ to the prolonged half-life of Mcl-1 via phosphorylation-mediated protein stability. These data corroborate our earlier findings linking O_2_^.−^-driven signaling to apoptotic inhibition [44], such as the constitutive Rac1 activation, phosphorylation-mediated activation of c-Myc, Bcl-2, Stat-3, and NF-κB [29, 32, 33, 35, 45–49].

The role of redox adaptation in mediating resistance to targeted and/or chemotherapy is well-documented [33, 47, 50–52]. While an overwhelming oxidative stress, such as chemotherapy-induced, triggers cancer cell execution [53, 54], a mild pro-oxidant milieu endows cells with a survival advantage [33, 47, 55, 56]. Corroborating the latter, a mild increase in ROS is essential for maintaining a transformed phenotype of cancer cells [55, 57]. This is vividly demonstrated by our current study, that a mild increase in intracellular O_2_^.−^ promotes Mcl-1 stability as a mechanism of VEN resistance acquisition. Interestingly, recent studies have identified that increased OXPHOS potential plays a critical role in determining sensitivity to Bcl-2 inhibition. These studies further demonstrated that the deregulated expressions of AMPK subunits, anti-apoptotic protein, and/or mitochondrial complex VI subunit as critical phenomena to metabolic reprogramming and the increase in OXPHOS potential in VEN-R cells [13, 52]. Given that our earlier work has suggested that increased OXPHOS could increase the probability of mitochondrial electron leakage onto oxygen to generate mitochondrial O_2_^.−^ [30, 35], these collective evidence provide a mechanistic hypothesis that deregulated expression of these proteins could be upstream players to the enhanced OXPHOS, mitochondrial O_2_^.−^ production, AKT activation, Mcl-1 phosphorylation and stability, which ultimately drives VEN resistance. Notably, VEN-induced pro-oxidant milieu was demonstrated by another study in which Nrf2 inhibition and mitochondrial ROS production were implicated [58]. It is, therefore, plausible that the sustained pro-oxidant milieu in VEN-R cells is an adaptation to oxidative stress upon prolonged VEN exposure. Alternatively, intracellular O_2_^.−^ generation in VEN-R cells could be due in part to outgrowth of *Ras*-mutant clones, as reported in AML patients treated with VEN-containing regimens [15]. In this regard, O_2_^.−^ has been implicated in oncogenic signaling upon Ras activation [59, 60].

While Mcl-1 expression is regulated at multiple cellular levels, redox-dependent increase in its stability in VEN-R cells is a function of post-translational modification and supports earlier findings that T163pMCL-1 promotes Mcl-1 stability [24, 25]. We highlight the importance of O_2_^.−^-induced T163pMcl-1 in maintaining the viability of VEN-R cells. Interestingly, the unstructured N-terminus of Mcl-1 promotes its proteasomal degradation, and several E3-ligases promote Mcl-1 degradation [61–63]. Ubiquitination and proteasomal degradation are facilitated by interaction with NOXA, whereas BIM binding stabilizes Mcl-1 [12, 64]. Although the E3-ligase regulation in VEN-R cells remains elusive, we demonstrated that increased O_2_^.−^ stabilizes Mcl-1 by reducing its ubiquitination, and by enhancing its binding to BIM.

Interestingly, O_2_^.−^-dependent AKT activation is linked to increased T163pMcl-1. AKT plays an important role in mediating survival and proliferation in AML cells [65, 66]. While AKT activation has been described in VEN-R cells [67], its association with altered intracellular redox environment, including O_2_^.−^-induced S473pAKT, T163pMcl-1, and stabilized Mcl-1, is novel. As to how O_2_^.−^ triggers AKT activation, a previous report demonstrated PTEN inactivation via S-nitrosylation as an underlying mechanism of AKT activation [38]. Whether this is involved in VEN-R cells remains to be explored. Notably, interaction between Mcl-1 PEST domain and pleckstrin homology (PH) domain of AKT has been reported [37]. The interaction between AKT and Mcl-1 is further supported by the fact that T163 resides in the PEST domain. Whether this physical interaction is involved or further enhanced in the context of VEN resistance, in conjunction with the potential involvement of redox-inactivation of PTEN and interaction between PTEN, AKT, and Mcl-1, remains an interesting topic to be addressed. AKT activation also explains the absence of S159pMcl-1, as GSK3β is inactivated upon AKT phosphorylation. Furthermore, as the PI3K/AKT pathway is frequently activated together with mutated FMS-related receptor tyrosine kinase (FLT3) in AML [65], it is not surprising that S159pMcl-1 is undetected in VEN-R cells. Finally, our work demonstrates that capivasertib prevented T163pMcl-1 and Mcl-1 stability and restored VEN sensitivity. Given that capivasertib has recently been approved as part of a treatment combination for breast cancer [39], and is being tested pre-clinically [68] and in relapsed and refractory (R/R) non-Hodgkin lymphoma patients (NCT05008055), increase the likelihood of utilizing this regimen due to its existing and potentially manageable patient safety profile as well as provide a strong rationale for its clinical utility in patients who have relapsed on or are refractory to VEN therapy.

In summary, while Mcl-1 upregulation has been a key feature in relapse and refractory patients treated with the FDA-approved VEN as well as in various experimental models of VEN resistance, there has yet to be a comprehensive study delineating the mechanism of this upregulation. Our finding addresses this gap by providing a clear mechanism of Mcl-1 stabilization and accumulation, which involves an increase in intracellular O_2_^.−^ level and subsequent AKT activation to promote T163 phosphorylation and stability of Mcl-1. As we showed that this novel resistance mechanism is conserved across different major hematologic malignancies, our study highlights actionable nodes for therapeutic intervention to restore sensitivity to VEN via modulating intracellular O_2_^.−^ and/or inhibiting AKT. Given the pressing issue of relapse and refractory cases in VEN-treated CLL and AML patients, our findings may ultimately impact treatment strategies and patient outcomes.

## Methods

### Western blot analyses

Western blot analysis was used to detect phosphorylation or total protein levels, as previously described [32, 33]. Precision Plus Protein^TM^ Kaleidoscope^TM^ prestained protein standards (Cat.#1610375, Bio-Rad, California, USA) were used. ImageJ was used for densitometric quantification of western blot bands.

### Plasmids, siRNAs, and cell transfection

*pcDNA3.1-MCL1*^*wt*^*, pcDNA3.1-MCL1*^*T163A*^ plasmids were gifts from Roger Davis (Addgene, #25375, #25376). *pcDNA3-HA-Ubiquitin* was a gift from Edward Yeh (Addgene, #18712). onTARGETplus human *AKT1 siRNA* smartpool (Cat.#L-003000-00), and onTARGETplus *SOD1 siRNA* sequences (Cat.#J-008364–09,J-008364–10) were purchased from Dharmacon Technologies, Cambridge, UK. Cells were transfected with 2 µg *pcDNA3.1, MCL1*^*wt*^*, MCL1*^*T163A*^ plasmids, or 100 nM *siRNA* using the Neon Electroporation system (Invitrogen, California, USA).

### Detection of ROS and O_2_^.−^

Detections of ROS and O_2_^.−^ were performed using the O_2_^.−^-sensitive lucigenin chemiluminescence assay, 2’,7’-dichlorofluorescein diacetate (DCFDA) assay, and MitoSOX^TM^ Red O_2_^.−^ assay, as described previously [46–48]. A detailed protocol is included as Supplementary Information.

### BH3-profiling technique

BH3-profiling was used to measure the priming of cells towards apoptosis and to determine the cellular dependency on specific anti-apoptotic proteins for survival [69]. A detailed protocol is included as supplementary information.

### Statistical analyses

Results are represented in graphs with mean ± SD. Two-tailed *T* test, one-way or two-way ANOVA, Sidak’s, Tukey’s, Dunnett’s, or Holm–Sidak’s multiple comparison test was used depending on the comparisons made. Pearson's correlation coefficient, *r*, was used to test for correlations between two variables. All experiments were repeated at least two times unless otherwise stated. Statistical significance was set at *P* < 0.05.

Other materials and methods could be found in the supplementary materials file.

## Supplementary information

Supplemental Figures and Legends

Supplemental Materials and Methods

## Data Availability

All materials and data are available from the corresponding authors upon reasonable request.
