# Supplementary material for: Superoxide-mediated phosphorylation and stabilization of Mcl-1 by AKT underlie venetoclax resistance in hematologic malignancies
Source: Leukemia. 2025 Jul 24;39(10):2477–91. doi: 10.1038/s41375-025-02694-4 (PMC12463670; doi:10.1038/s41375-025-02694-4)
Supplement: Supplementary file 1 — Supplemental Figures and Legends [file 41375_2025_2694_MOESM1_ESM.pdf]

Supplemental Figures

Supplemental Figure 1. VEN-R MOLM14 cells are resistant to VEN treatment.

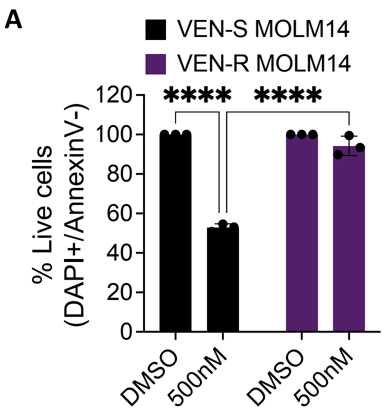

A) Bar graph showing DAPI/Annexin V assay measurement of % live VEN-S and VEN-R MOLM14 cells following treatment with VEN (500nM/0.5μM) for 48 hours. N=3. Sidak's multiple comparisons test was used.

Supplemental Figure 2. VEN treatment increases ROS levels in VEN-S MOLM14 cells.

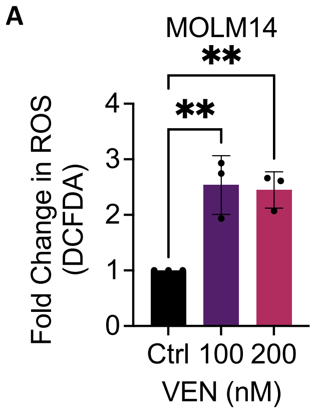

A) Bar graph showing fold change in ROS by DCFDA staining following 48-hour treatment with VEN (100 - 200nM/0.1 - 0.2μM) in MOLM14 cells. N=3. Dunnett's multiple comparisons test was used.

Supplemental Figure 3. $O_2^{\cdot-}$ mediates Mcl-1 upregulation in various hematologic malignancies.

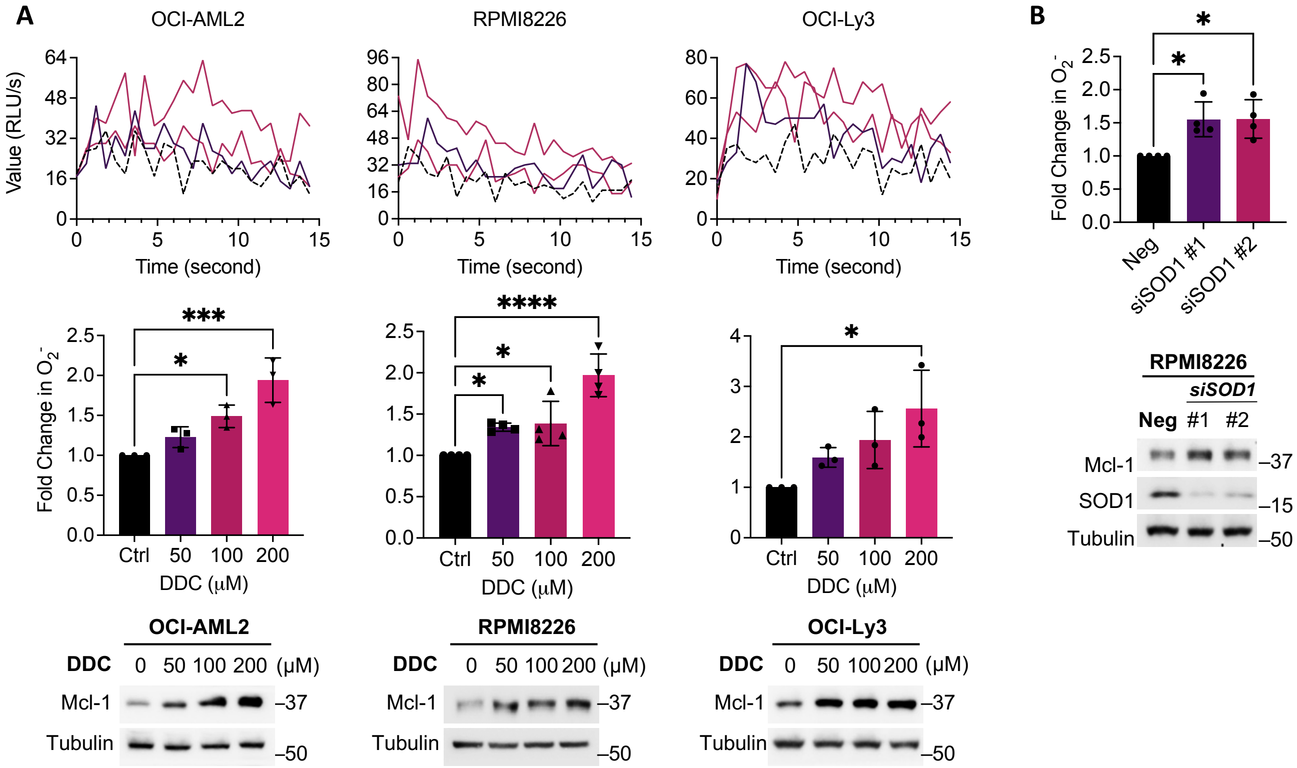

A) Line graphs showing raw $O_2^{\cdot-}$ detection following treatment with DDC (50 - 200μM) in OCI-AML2, RPMI8226 and OCI-Ly3 cells for 4 hours. Bar graph showing fold change of averaged $O_2^{\cdot-}$ levels following DDC treatment. Western blot showing Mcl-1 and β-Actin levels following treatment with DDC (50 - 200μM) in OCI-AML2 (N=3), RPMI8226 (N=4) and OCI-Ly3 (N=3) cells for 4 hours. Holm-Sidak's multiple comparisons test was used.

B) Bar graph showing fold change of averaged $O_2^{\cdot-}$ levels following *siSOD1* treatment for 48 hours in RPMI8226 cells. Western blot showing Mcl-1 and β-Actin levels following treatment with *siSOD1*. N=4. Holm-Sidak's multiple comparisons test was used.

Supplemental Figure 4. $O_2^{\cdot -}$ protects cells from VEN and S63845-induced apoptotic cell death.

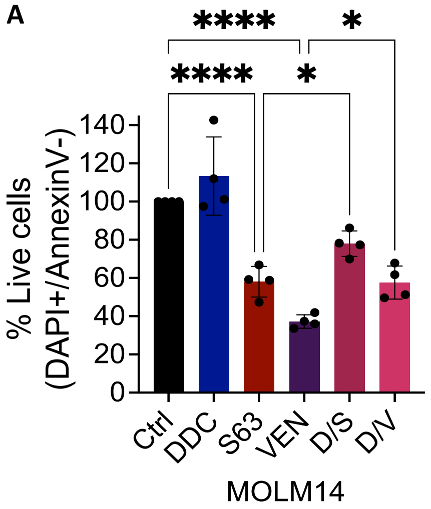

A) Graph showing DAPI/Annexin V assay measurement of % live VEN-S MOLM14 following pre-treatment with DDC (25μM) for 2 hours and co-treatment with VEN (0.1μM) or S63845 (0.1μM) for 48 hours. N=4. S63 – S63845, D/S – DDC and S63845, D/V – DDC and VEN. Sidak's multiple comparisons test was used.

Supplemental Figure 5. VEN treatment increases ROS and Mcl-1 levels in VEN-R MOLM14 cells.

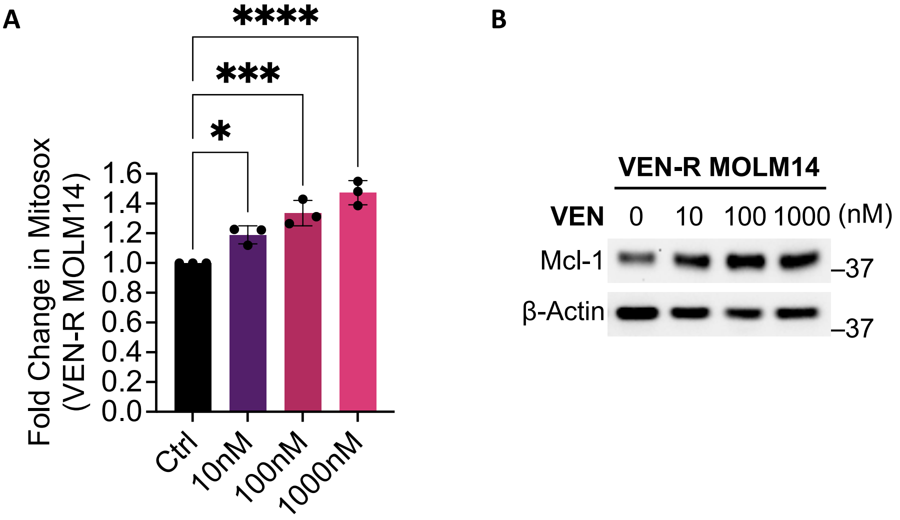

A) Bar graph showing fold change of mitochondrial $O_2^{\cdot -}$ levels following treatment with increasing concentration of VEN (10 – 1000nM/0.01 – 1μM) in VEN-R MOLM14 cells for 24 hours. N=3. Dunnett's multiple comparisons test was used.

B) Western blot showing Mcl-1 and β-Actin levels following treatment with increasing concentration of VEN (10 – 1000nM) in VEN-R MOLM14 cells for 24 hours. N=3.

Supplemental Figure 6. Scavenging $O_2^{\cdot-}$ re-sensitizes VEN-R cells to VEN-induced apoptotic cell death.

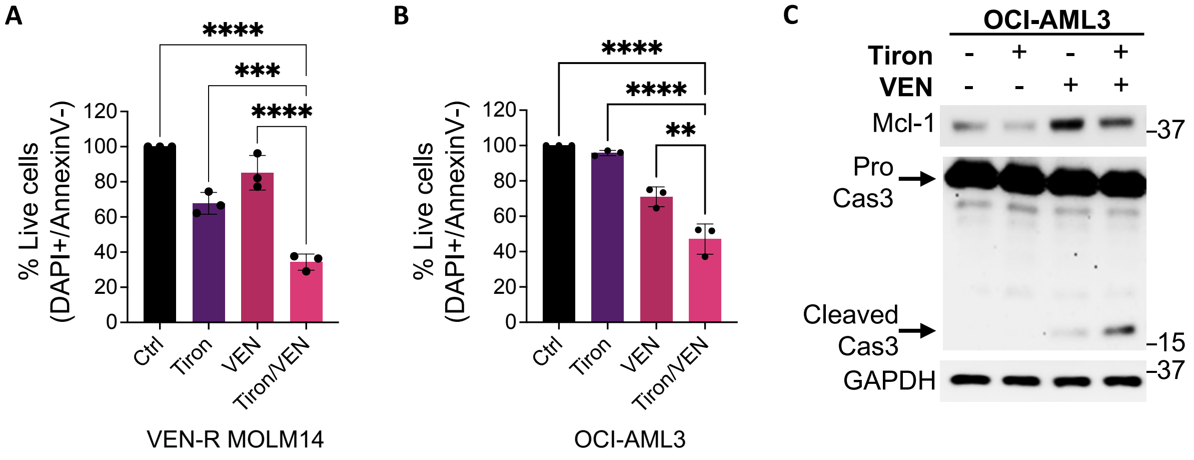

A-B) Bar graph showing DAPI/Annexin V assay measurement of % live VEN-R MOLM14 and OCI-AML3 cells following pre-treatment with Tiron (2.5mM or 1mM respectively) for 2 hours and co-treatment with VEN (0.1μM or 0.5μM respectively) for 48 hours. N=3. Sidak's multiple comparison test was used.

C) Western blot showing Mcl-1, pro and cleaved caspase-3, GAPDH levels of OCI-AML3 cells following pre-treatment with $O_2^{\cdot-}$ scavenger, Tiron (1mM) for 2 hours followed by VEN (0.5μM) for 48 hours. N=3.

Supplemental Figure 7. $O_2^{\cdot-}$-mediated Mcl-1 upregulation is not due to increased *MCL1* gene transcription.

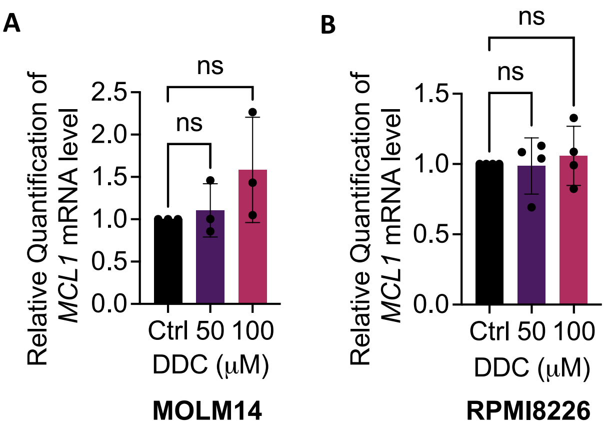

A-B) Graph showing relative quantification of *MCL1* mRNA levels of MOLM14 (N=3) or RPMI8226 (N=4) cells treated with indicated doses of DDC (μM) for 4 hours. Data were normalized to untreated control. Holm-Sidak's multiple comparisons test was used.

Supplemental Figure 8. $O_2^{\cdot -}$ regulates T163pMcl-1 and total Mcl-1.

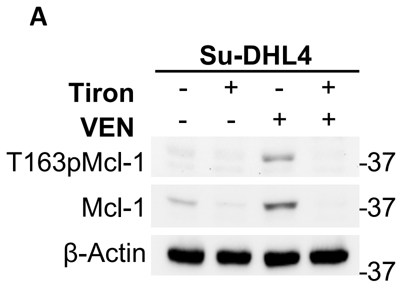

A) Western blot showing T163pMcl-1, Mcl-1 and β-Actin levels of Su-DHL4 cells treated with Tiron (5mM) for 2 hours followed by VEN (0.5μM) for 24 hours. N=3.

Supplemental Figure 9. VEN-R cells are highly dependent on Mcl-1 for survival.

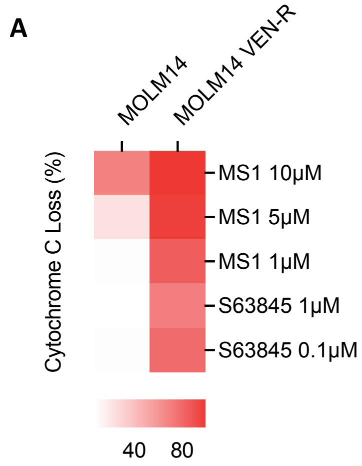

A) Heatmap of BH3-profiling showing percentage cytochrome *c* loss from VEN-S MOLM14 or VEN-R MOLM14 cells following incubation with MS1 peptide or S63845 drug. N=3.

Supplemental Figure 10. $O_2^{\cdot-}$-mediated Mcl-1 upregulation is mediated by AKT.

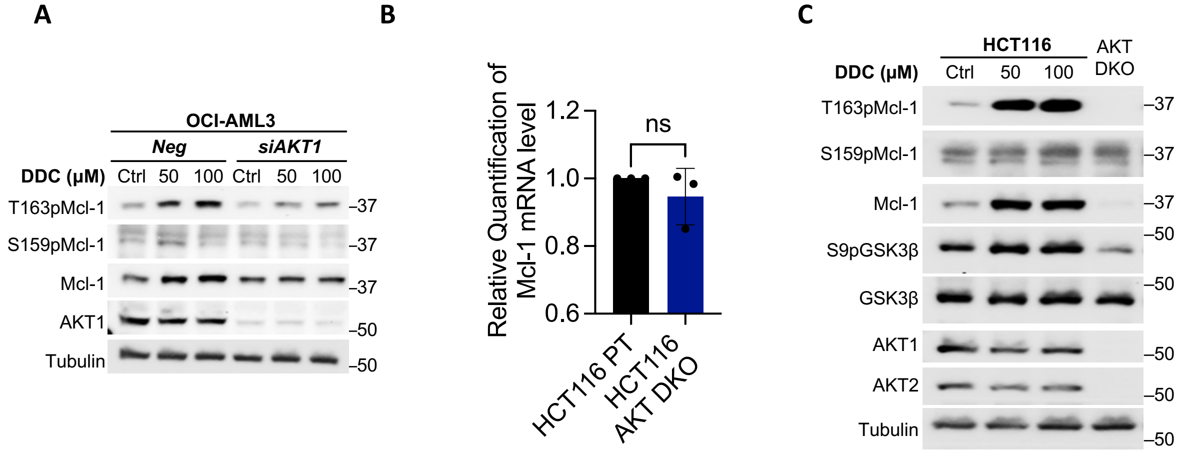

A) Western blot showing T163pMcl-1, S159pMcl-1, Mcl-1, AKT1 and tubulin levels of OCI-AML3 cells transfected with *Neg* or *siAKT1* for 48 hours before treatment with DDC (50-100uM) for 4 hours.

B) Graph showing relative quantification of *MCL1* mRNA levels of HCT116 parental (PT) cells vs AKT double knockout (*AKT*-DKO) cells. Data were normalized to HCT116 parental cells. Unpaired T test was used.

C) Western blot showing T163pMcl-1, S159pMcl-1, Mcl-1, S9pGSK3β, GSK3β, AKT1, AKT2 and tubulin levels in HCT116 PT cells treated with DDC (50 - 100μM) for 4 hours and HCT116 AKT-DKO cells.

Supplemental Figure 11. VEN resistant cells display increased phospho-activated AKT.

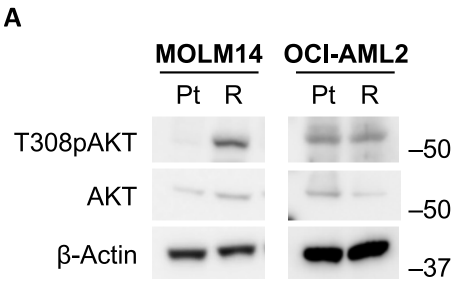

A) Western blot showing T308pAKT, AKT and β-Actin levels in resistant and parental MOLM14 or OCI-AML2 cells.

Supplemental Figure 12. AKT inhibition re-sensitizes VEN-R cells to VEN-induced cell death.

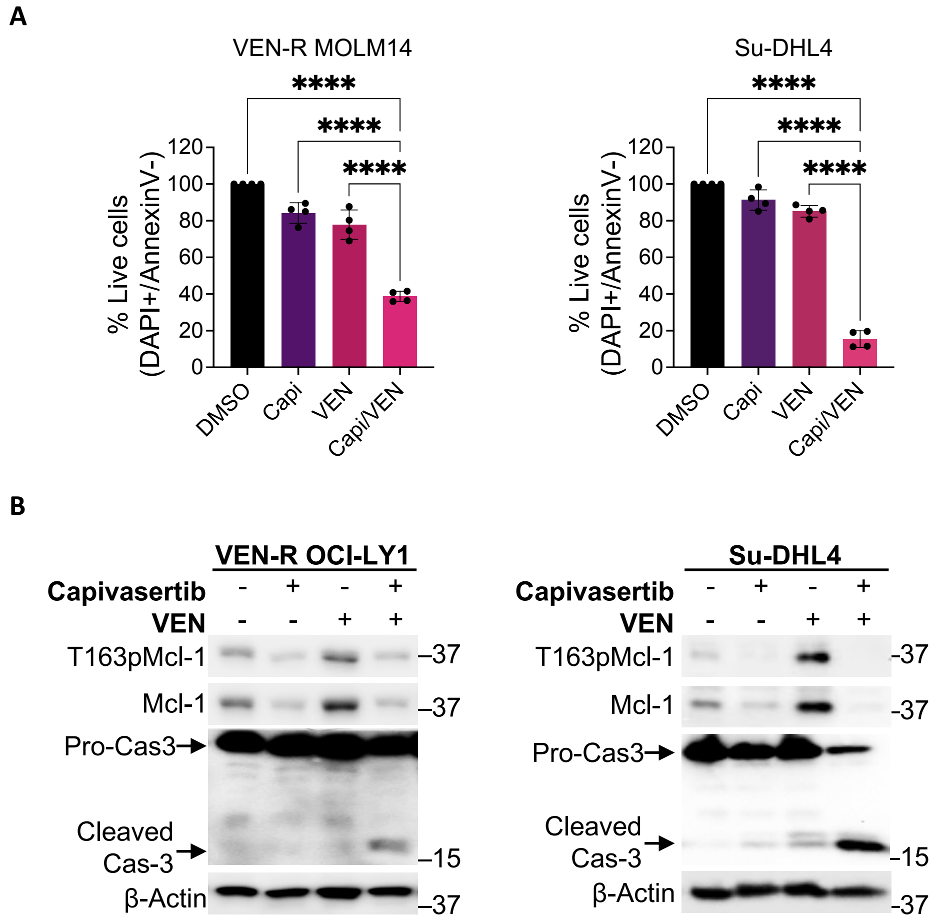

A) Graph showing DAPI/Annexin V assay measurement of % live VEN-R MOLM14 and Su-DHL4 cells treated with capivasertib (5μM/MOLM14 VEN-R, 2μM/Su-DHL4) and/or VEN (0.1μM/MOLM14 VEN-R, 0.5μM/Su-DHL4) for 48 hours. N=4. Sidak's multiple comparisons test was used.

B) Western blot showing T163pMcl-1, Mcl-1, pro and cleaved caspase 3, β-Actin levels in VEN-R OCI-Ly1 or Su-DHL4 cells treated with capivasertib (0.5μM/OCI-Ly1 VEN-R, 2μM/Su-DHL4) and/or VEN (1μM/OCI-Ly1 VEN-R, 0.5μM/Su-DHL4) for 24 hours. N=3.

Supplemental Figure 13. AKT inhibition reduces Mcl-1 levels in primary CLL cells.

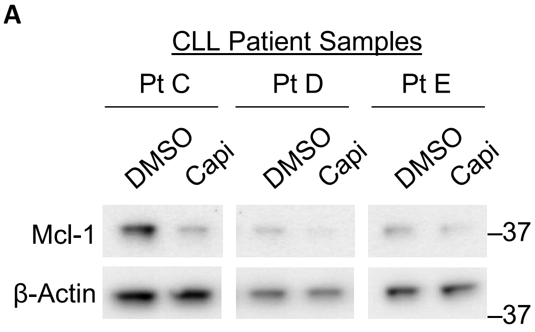

A) Western blot showing Mcl-1 and β-Actin levels following treatment with capivasertib (2μM) or DMSO for 6 hours in CLL patient samples.

Supplemental Figure 14. AKT inhibition induced minimal reduction in S70pBcl-2

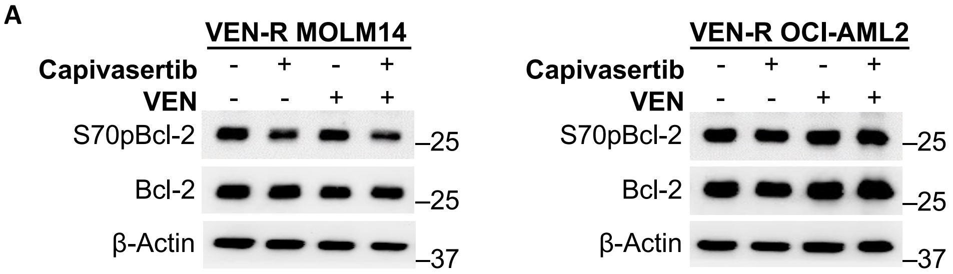

A) Western blot showing S70pBcl-2, Bcl-2 and β-Actin levels in VEN-R MOLM14 or VEN-R OCI-AML2 cells treated with capivasertib (1μM/MOLM14 VEN-R, 5μM/Su-DHL4) and/or VEN (0.1μM/MOLM14 VEN-R, 10μM/OCI-AML2) for 24 hours. N=3.

Supplemental Figure 15. Treatment schedule for VEN-R MOLM14 xenograft model

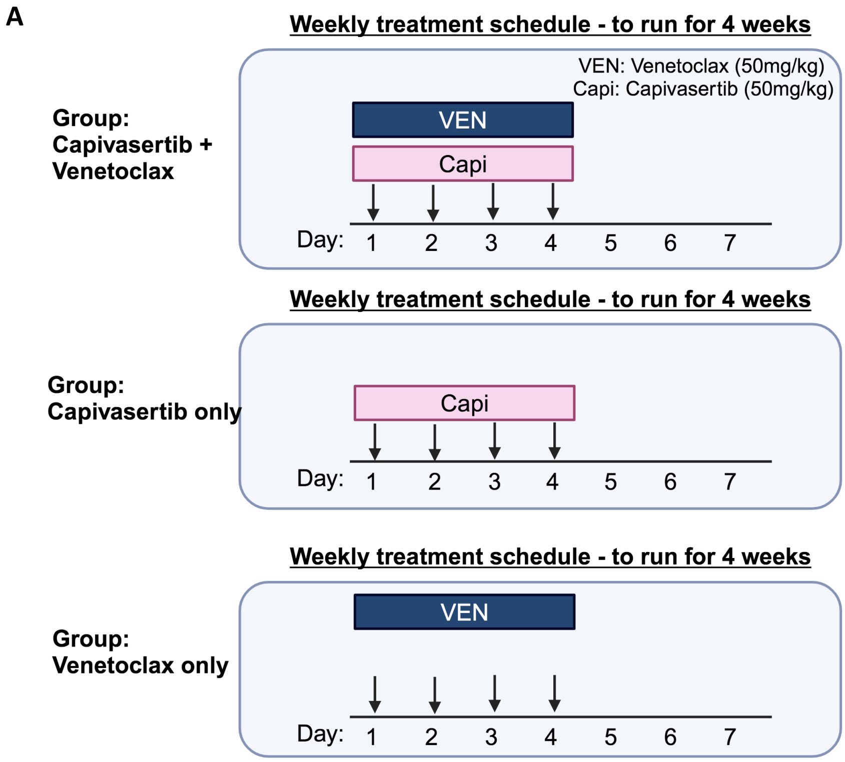

A) Diagram showing treatment schedule for female NRG-SGM3 mice harbouring VEN-R MOLM14 cells. 2 millions cells were injected into mice through tail vein, randomized and incubated for 14 days prior to the measurement of tumor burden from blood sampling by flow cytometry and initiation of treatment based on the schedule (Numbering for day on diagram indicates day of the week, *i.e.* 1 is Monday). Treatment runs for 4 weeks. Tumor burden were measured from blood samples every first day of the week through flow cytometry. Survival of mice was recorded based on specified endpoints such as immobility or hindleg paralysis, inability to feed and/or lack of grooming. Diagram was Created in BioRender. Pervaiz, S. (2024) BioRender.com/z38m010.
