# Supplementary material for: Superoxide-mediated phosphorylation and stabilization of Mcl-1 by AKT underlie venetoclax resistance in hematologic malignancies
Source: Leukemia. 2025 Jul 24;39(10):2477–91. doi: 10.1038/s41375-025-02694-4 (PMC12463670; doi:10.1038/s41375-025-02694-4)
Supplement: Supplementary file 2 — Supplemental Materials and Methods [file 41375_2025_2694_MOESM2_ESM.pdf]

__Supplementary Information:__

Detailed Materials and Methodology

A) *Cell lines and culture conditions*

Human Jurkat (ATCC® TIB-152™) was obtained from ATCC, Virginia, USA. RPMI8226, MOLM14, OCI-AML2, OCI-AML3 cells were from the Chng Lab, Cancer Science Institute (CSI), Singapore. TMD8, Su-DHL4, OCI-Ly1, VEN-R OCI-Ly1 cells were from the Wu Lab, Dana-Farber Cancer Institute (DFCI), MA, USA. OCI-Ly3 and stroma NKTert cells were from the Davids Lab, DFCI. Cells were cultured in RPMI1640 supplemented with 10% FBS, 1% penicillin-streptomycin, and 1% L-glutamine. OCI-AML2, OCI-AML3 cells were specifically cultured in MEMα supplemented with 20% FBS. Jurkat CRISPR/Cas9 Mcl-1 knockout cells were generated using the '*MCL1 CRISPR/Cas9 KO'* (sc-400079) and 'HDR' (sc-400079-HDR) plasmids. To generate VEN-R cells, MOLM14 or OCI-AML2 cells were continuously cultured in incremental doses of venetoclax (ABT199) over a period of 10 weeks until the emergence of clone(s) resistant to 1μM venetoclax. For *ex vivo* treatment using primary CLL cells, stroma NKTert cells were plated one day before seeding the primary CLL cells for respective treatments and subsequent Annexin-V assay, as previously described[1]. Cells were cultured at 37°C in a humidified atmosphere of 95% air and 5% CO_2. Cells used in this study were either of early passages, recently purchased or authenticated by STR profiling and were routinely checked for the absence of mycoplasma.

B) Generation of Mcl-1 knockout cells

Jurkat cells were transfected with 1.5μg of both *MCL1 KO* and HDR plasmids via electroporation. Successfully transfected cells were selected with 0.75μg/ml of puromycin and sorted into single cell clones. *MCL1 KO* was verified in Jurkat cells by western blotting and the monoclonal cells were sub-cultured in RPMI1640 supplemented with 10% FBS.

C) Chemicals and reagents

Fetal Bovine Serum (FBS) and Phosphate Buffered Saline (PBS) were purchased from Hyclone, Thermofisher Scientific, Waltham, MA, USA. 3-(4,5-dimethylthiazol-2-yl)-2,5-diphenyltetrazolium bromide (MTT), diethyldithiocarbamate (DDC), 4,5-dihydroxy-1,3-benzenedisulfonic acid (Tiron), diphenyleneiodonium (DPI), PD98059, LY294002, N,N′- Dimethyl-9,9′-biacridinium dinitrate (Lucigenin), dimethyl sulfoxide (DMSO), phenylmethylsulfonyl fluoride (PMSF), sodium fluoride (NaF), dithiotheritol (DTT), ammonium persulphate (APS), tetramethylethylenediamine (TEMED), Ponceau S were purchased from Sigma Aldrich, St. Louis, MO, USA. Goat anti-mouse and anti-rabbit IgG horseradish peroxidase conjugated secondary antibodies were purchased from Pierce, TX, USA. Venetoclax/ABT199, S63845, and capivasertib (AZD5363) were purchased from Selleck Chemicals, TX, USA or MedChemExpress, NJ, USA. Protein A agarose beads (#sc-2001) were purchased from Santa Cruz, TX, USA.

Antibodies

Mcl-1 (Cat.#SC-12756) mouse and AKT1 (Cat.#SC-5298) mouse primary antibodies were purchased from Santa Cruz, TX, USA. T163pMcl-1 (Cat.#14765) rabbit, S70pBcl2 (Cat.#2827) rabbit, S473pAKT (Cat.#4060S) rabbit, Mcl-1 (Cat.#39224) rabbit, AKT2 (Cat.#3063S) rabbit, T202/Y204pMAPK (Cat.#4370S) rabbit, ERK1/2 (Cat.#9102S) rabbit, S9pGSK3β (Cat.#9322S) rabbit, GSK3β (Cat.#9315S) rabbit, caspase-3 (Cat.#9662S) rabbit, GAPDH (Cat.#2118S) rabbit, α-Tubulin (Cat.#2125S) rabbit, BIM (Cat.#2819) rabbit primary and Lys48 (K48)-linkage specific polyubiquitin rabbit (Cat.#8081) antibodies were purchased from Cell Signaling Technologies, MA, USA. S159pMcl-1 (Cat.#Ab28147) rabbit primary antibody was purchased from Abcam. Bak (Cat.#556382) mouse, SOD1 (Cat.#G215-1) mouse, Mcl-1 (Cat.#556547) mouse and Bcl-2 (Cat.#610539) mouse primary antibodies were purchased from BD Bioscience, MA, USA. hCD45-FITC (Cat.#982316) and hCD33-PE (Cat.#303404) antibodies were purchased from Biolegend, CA, USA.

For performing immunoprecipitation (IP) assays, primary antibodies against Mcl-1 (Cat.#16225-1-AP) was purchased from ProteinTech, Rosemont, Illnois, USA. BIM (Cat.#SC-374358) and Mcl-1 (Cat.# sc-12756) were purchased from Santa Cruz.

D) Patient samples preparation

Lymphoma biopsies received were grinded by two sterile glass slides in RPMI medium and strained through a MACS filter to obtain single cell suspension. Cell suspension was centrifuged and cell pellet was lysed in either RIPA (supplemented with protease and phosphatase inhibitors; 10 µg/ml aprotinin, 10 µg/ml leupeptin, 20 µg/ml pepstatin A, 1 mM NaF, 1 mM Na_3VO_4 and 1 mM PMSF) and stored at -80°C before subsequent analysis. Isolation of primary CLL cells were performed as previously described[1].

E) Determination of cell viability

Cell viability was assessed using (3-(4,5-dimethylthiazol-2-yl)-2,5-diphenyltetrazolium bromide (MTT) method as described previously[2,3]. CellTitre-Glo (CTG) (Cat.#PR-G7573, Promega) was used according to manufacturer's protocol. For Annexin-V staining, detailed protocol is included as supplementary information. For Annexin-V staining assay, cells were stained with 15µl of 10× Annexin-V binding buffer (100 mM HEPES, 40 mM KCl, 1.4 M NaCl, 7.5 mM $MgCl_2$, 25 mM $CaCl_2$ pH 7.4) with Annexin-V (Cat.#556547, BD Biosciences) and Hoechst-33342 (Cat.#H3570, Invitrogen) for 15min at RT. Subsequently fixed with fixing buffer (4% formaldehyde, 0.5% glutaraldehyde in 1× Annexin-V binding buffer) for 10 min and neutralized with N2 buffer (1.7 M Tris, 1.25 M glycine, pH9.1). Samples were gated and analyzed based on live population (Hoechst$^+$ Annexin V$^-$) using Cytoflex LX flow cytometer (Beckman Coulter, IN, USA) as described by Villalobos-Ortiz *et al*[4].

F) Detection of intracellular and mitochondrial $O_2^{\cdot -}$

For intracellular $O_2^{\cdot -}$, cells were resuspended in 450µl of ATP lysis buffer. 400µl of cell suspension was used to measure intracellular $O_2^{\cdot -}$ levels using the Berthold Sirius luminometer. Luminescence

signal obtained was measured as relative light units (RLU) every 0.6 second up until 14.4th second. Data were analyzed by averaging all RLU data points and normalized to protein concentration (quantified with the leftover 50µl of lysate). Results were presented as fold change in RLU/µg of protein, normalized to untreated cells.

For mitochondrial $O_2^{\cdot -}$ and general intracellular ROS, the MitoSOX™ (Cat.#M36008, Invitrogen) and DCFDA (Cat.#C6827, Invitrogen) were used, respectively. Cells (0.2 x 10^6 cells) were washed with PBS and incubated with 5µM Mitosox or 5µM DCFDA in plain/FBS-free RPMI for 15 minutes at 37°C. Samples were further diluted with 500µL of RPMI prior to measurement with the Cytoflex LX flow cytometer (Beckman Coulter). At least 10 000 events were analyzed.

G) Oxygen consumption rate (OCR)

Cellular OCR (0.5 x 10^6 cells) was measured using the XF24 Analyzer (Seahorse Bioscience, MA, USA) according to manufacturer's instructions. FCCP was used to obtain measurements for maximal respiration rate. Rotenone and antimycin A were used to measure mitochondrial OCR.

H) Real-time PCR analysis

RNA concentration was quantified using the Nanodrop (Nanophotometer N60, Implen, Munich, Germany). After obtaining RNA concentrations, 2µg of RNA was reverse transcribed to cDNA via the reverse transcription kit (Onescript Plus cDNA synthesis kit, Applied Biological Materials Inc, BC, Canada) according to manufacturer's instruction. Next, cDNA was mixed with SYBR Select master mix (Applied Biosystems, MA, USA) according to manufacturer's instructions. qRT-PCR was performed on Lightcycler 480 Instrument (Roche). Sequences of the primers used in this study are listed below:

Mcl-1 Forward: *5'-GTGCCTTTGTGGCTAAACACT-3'*

Mcl-1 Reverse: *5'-AGTCCCGTTTTGTCCTTACGA-3'*

β-actin Forward: *5'-CACCATTGGCAATGAGCGGTTC-3'*

β-actin Reverse: *5'-AGGTCTTTGCGGATGTCCACGT-3'*

I) BH3 profiling

5 million primary CLL cells isolated from the peripheral blood or 3 million cells of immortalized cell line were suspended in 1.65mL MEB2P buffer (150 mM mannitol, 10 mM HEPES-KOH pH 7.5, 150 mM KCl, 1 mM EGTA, 1 mM EDTA, 0.1% BSA, 5 mM succinate, 0.25% poloxamer 188). Before transferring 15μL of the cell suspension to the BH3 profiling 384-well plate coated with 0.002% digitonin for cell membrane permeabilization, increasing concentrations of BIM, BAD, PUMA, HRK and MS1 11-mer pro-apoptotic peptides as well as BH3 mimetic ABT199 (venetoclax). Cells were incubated in this plate for 1 hour and thereafter fixed with 15μL of 4% paraformaldehyde for 30 minutes followed by neutralization with 15μL of N2 buffer (1.7 M Tris, 1.25 M Glycine pH 9.1) for 20 minutes. Cells were then stained with 10μL of a staining cocktail consisting of PE-Cy7-conjugated CD19 (Cat.#302216, Biolegend), PE-conjugated CD5 (#300608, Biolegend), Alexa-488-conjugated cytochrome *c* (Cat.#612308, Biolegend) antibodies and Hoechst 33342 (Cat.#H3570, Invitrogen) and incubated overnight prior to analysis on the BD FACS Fortessa flow cytometry. Cells were gated using Hoechst as live cells, CD5 and CD19 for CLL primary cell surface markers for the Alexa-488-conjugated cytochrome *c* signal. Another cocktail without PE-Cy7-conjugated CD5 and PE-conjugated CD19 was used for cell lines. For Dynamic BH3 profiling (DBP), cells were treated with drug-of-interest prior to initiating the BH3 profiling as aforementioned. Cytochrome *c* release was measured as the degree of MOMP induction to identify anti-apoptotic protein dependences in response to the corresponding pro-apoptotic peptide after the normalization to 0% cytochrome *c* release by DMSO (negative control) and 100% cytochrome *c* release by alamethicin (positive control). Induction/increase in cytochrome *c* release indicates specific dependencies for certain anti-apoptotic protein. HRK and A1331852 were used for BCL-xL dependency, MS1 and S63845 for MCL-1, BAD for BCL-2 and/or BCL-xL, venetoclax for BCL-2. BIM and PUMA were used as overall mitochondrial priming indicator for apoptosis.

J) Immunoprecipitation assay

Briefly, cells were harvested and lysed in immunoprecipitation (IP) lysis buffer (#cat. 87788, ThermoFisher Scientific) supplemented with protease and phosphatase inhibitors (#cat. 539136, 524625, Milipore). Cells in IP lysis buffer were then snap-frozen and used for subsequent IP experiment. Protein concentration were measured from thawed samples using Bradford's assay. 1500μg of protein per sample was isolated for pre-clearing using 15μL protein agarose G beads (#cat. Sc-2002, Santa Cruz) for 1 hour on a rotator at 4°C. Samples were then centrifuged and supernatants were transferred to new 1.5ml tubes. 3μg of MCL-1 antibody (#cat. sc-12756) was added in each sample and incubated on rotator at 4°C overnight. Subsequently, 25μL protein agarose G beads were added to each sample for pull down on a rotator at 4°C for 5 hours. Samples were then washed 3 times with 400μL IP buffer. 25μL of loading dye were added to each sample and heated at 95°C for 15 minutes before undergoing western blot analysis. For IP experiment related to ubiquitination, Lys48 (K48)-linkage specific polyubiquitin antibody (#cat. 8081, Cell Signaling) was used for immunoblotting of immunoprecipitated Mcl-1. For Lys48-Mcl-1 IP, we first pre-treated 15 million cells with the proteasomal inhibitor 5μM of MG132 for 1 hour and subsequently treated with 100μM DDC for 4 hours.

K) Animal study

In-house bred 8-10 weeks old female NRG-SGM3 (EN3W) mice were used for *in vivo* animal study. 2 millions cells were injected into mice through tail vein, randomized and incubated for 14 days prior to the measurement of tumor burden from blood sampling by flow cytometry (CD45+ and CD33+ gating) and initiation of treatment. No blinding was applied for animal study. Treatments are divided into 4 arms, each arm with 4 mice. Treatment includes vehicle (DMSO), capivasertib (50mg/kg), venetoclax (50mg/kg) and combo. Treatment runs for 4 weeks. Tumor burdens (hCD45+ hCD33+ gating) were measured from blood sampling every first day of the week through flow cytometry. Survival of mice was recorded based on specified endpoints such as immobility or hindleg paralysis, inability to feed and/or lack of grooming.

L) Monitor circulating AML cells in mouse peripheral blood

Approximately 20–50μL of blood was collected from the facial submandibular vein using a surgical lancet and transferred into a microtainer blood collection tube. For red blood cell lysis, 10μL of blood was incubated with 300μL of ACK lysing buffer (Gibco) for 10 minutes at room temperature. The cells were washed with 2mL of PBS and centrifuged at 500 x g for 5 minutes at 20–22°C. After centrifugation, the cell pellet was resuspended in FACS staining buffer and stained with hCD45-FITC and hCD33-PE antibodies at a 1:100 dilution. Flow cytometry was performed to determine the percentage of circulating human myeloblasts, defined by gating on hCD45+ hCD33+ cells.

Additional References

1. Chong SJF, Zhu F, Dashevsky O, et al. Hyperphosphorylation of BCL-2 family proteins underlies functional resistance to venetoclax in lymphoid malignancies. *J Clin Invest*. 2023;133(22).

2. Chong SJF, Lai JXH, Qu J, et al. A feedforward relationship between active Rac1 and phosphorylated Bcl-2 is critical for sustaining Bcl-2 phosphorylation and promoting cancer progression. *Cancer Letters*. 2019;457:151-167.

3. Low ICC, Loh T, Huang Y, Virshup DM, Pervaiz S. Ser70 phosphorylation of Bcl-2 by selective tyrosine nitration of PP2A-B56δ stabilizes its antiapoptotic activity. *Blood*. 2014;124:2223-2234.

4. Villalobos-Ortiz M, Ryan J, Mashaka TN, Opferman JT, Letai A. BH3 profiling discriminates on-target small molecule BH3 mimetics from putative mimetics. *Cell Death Differ*. 2020;27(3):999-1007.
